# Dietary High Zinc Oxide Modulates the Microbiome of Ileum and Colon in Weaned Piglets

**DOI:** 10.3389/fmicb.2017.00825

**Published:** 2017-05-09

**Authors:** Ting Yu, Cui Zhu, Shicheng Chen, Lei Gao, Hang Lv, Ruowei Feng, Qingfeng Zhu, Jinsong Xu, Zhuang Chen, Zongyong Jiang

**Affiliations:** ^1^Agro-biological Gene Research Center, Guangdong Academy of Agricultural SciencesGuangzhou, China; ^2^Department of Microbiology and Molecular Genetics, Michigan State University, East LansingMI, USA; ^3^Key Laboratory of Crops Genetics and Improvement of Guangdong ProvinceGuangzhou, China; ^4^Ministry of Agriculture Key Laboratory of Animal Nutrition and Feed Science (South China), Guangdong Public Laboratory of Animal Breeding and Nutrition, Institute of Animal Science, Guangdong Academy of Agricultural SciencesGuangzhou, China

**Keywords:** zinc oxide, weaned piglets, intestinal microbiome, ileum, colon

## Abstract

Dietary zinc oxide (ZnO) at pharmacological level has been widely used to prevent and treat diarrhea in weaning piglets. Despite its importance for promoting animal health and performance, the influence of microbiome profiles in intestinal tracts by ZnO needs to be comprehensively investigated. In this study, we conducted a comparative microbial community analysis in the ileum and colon of piglets fed by either control diet, high ZnO (3,000 mg/kg) supplement or antibiotics (300 mg/kg chlortetracycline and 60 mg/kg colistin sulfate) supplement. Our results showed that both high dietary ZnO and in-feed antibiotics supplementations significantly increased 5 phyla of *Spirochaetes, Tenericutes, Euryarchaeota, Verrucomicrobia*, TM7, and reduced 1 phyla of *Chlamydiae* in ileal digesta. The relative abundance of opportunistic pathogens *Campylobacterales* were decreased while *Enterobacteriales* were increased in ZnO or antibiotics-supplemented group when compared to the control. In the colon, the phyla *Euryarchaeota*, the genus *Methanobrevibacter*, and the species *Methanobrevibacter smithii* were drastically increased by high dietary ZnO supplementation when compared with other groups. The microbial functional prediction analysis showed that high dietary ZnO and in-feed antibiotics had a higher abundance of transporter pathway enrichment in the ileum when compared with the control. While in the colon high dietary ZnO had a higher abundant enrichment of methane metabolism involving energy supply when compared with other groups. Both high dietary ZnO and antibiotics increased the microbiota diversity of ileal digesta while they decreased the microbiota diversity of the colonic digesta. Collectively, these results suggested that dietary ZnO and in-feed antibiotics supplementations presented similar effect on ileal microbiota, and mainly affected the non-predominant microbiota.

## Introduction

The piglets at early weaning stage are subjected to nutritional, physiological, and psychological stressors, leading to post-weaning diarrhea (PWD) and consequent growth retardation (Pluske et al., 1997; Lallès et al., 2007). In-feed antibiotics are often necessary to alleviate the PWD. However, in-feed antibiotics have been strictly regulated in the application of modern livestock industry due to the increase of antibiotic resistance and its potential contaminations to human (Barton, 2000; Bach Knudsen, 2001; Smith et al., 2002). Instead, dietary supplements with zinc oxide (ZnO) will be more promising because it contributes to better growth performance (Broom et al., 2006; Namkung et al., 2006; Zhu et al., 2017), enhancement of intestinal morphology and function, improvement in antioxidant capacity as well as restorations of the mucosal barrier integrity in weaned piglets (Sargeant et al., 2010; Zhu et al., 2017). The underlying mechanism of high ZnO on the regulation of intestinal health in weaned piglets may have an impact on the gut microbiome. However, the effects of high ZnO on the intestinal microbiome are somewhat controversial (Hojberg et al., 2005; Vahjen et al., 2010; Pieper et al., 2012; Starke et al., 2014). For instance, high ZnO was assumed to reduce the incidence of diarrhea by lowering the counts of pathogenic bacteria (Sales, 2013), while some studies have showed that ZnO increased the population of *Enterobacteriales* (Hojberg et al., 2005; Pieper et al., 2012). Moreover, one previous study done by analyzing the 16S rRNA sequencing showed that high dietary ZnO intake had a major impact on the porcine ileal bacterial compositions (Vahjen et al., 2010), where the lactic acid bacteria ratio was decreased (Vahjen et al., 2011). However, another study by denaturing gradient gel electrophoresis (DGGE) showed that high dietary ZnO supplementation resulted in a significant increase in most bacterial diversity in the ileum while no influence on lactic acid bacteria was observed (Pieper et al., 2012).

The inconsistent findings on the impacts of dietary ZnO affecting bacterial compositions were possibly caused by (1) the doses and duration of ZnO supplemented to the diets, (2) the different samples from various locations of porcine gastrointestinal tracts (stomach, small intestine, or large intestine), (3) the different methods (culturing, PCR, DGGE or deep sequencing) used for analyzing changes in the microbial community, and (4) the dynamic microbial community in the gastrointestinal tracts (Vahjen et al., 2010, 2011; Pieper et al., 2012; Sales, 2013; Starke et al., 2014). In order to investigate how ZnO impacts the intestinal health in piglets, we quantified the diversity of the bacterial community in the ileal and colonic digesta. Moreover, because ZnO was utilized as an alternative bactericide, we further compared bacterial communities to antibiotics chlortetracycline and colistin sulfate when they were provided in the diets for weaned piglets. The microbial functions were further predicted to explain how dietary high ZnO impacted the microbiota and improved intestinal homeostasis for alleviating the weaning stress of piglets. Thus, we hypothesized that dietary high ZnO might modulate the microbiome of ileum and colon to further influence the intestinal health of weaned piglets, and might partly exhibit similar effects as in-feed antibiotics.

## Materials and Methods

### Animals

The experimental protocols were approved by the Animal Care and Use Committee of Guangdong Academy of Agricultural Sciences, China. A total of ninety-six crossbred barrows (Duroc × Landrace × Large White) weaned at 21-day-old, with an initial body weight (BW) of 5.99 ± 0.04 kg, were used in this study, as described in our recent study (Zhu et al., 2017). Briefly, piglets were randomly allotted to three dietary treatment groups: (1) control group (piglets were fed basal diet without any in-feed antibiotics); (2) antibiotic group (piglets were fed the basal diet with in-feed antibiotics of 300 mg/kg chlortetracycline and 60 mg/kg colistin sulfate); and (3) ZnO group (piglets were fed the basal diet with 3,000 mg/kg ZnO). Each group had eight replicates (pens) with four piglets per replicate. The basal diets (Supplementary Table S1) were formulated to meet nutrient requirements for piglets recommended by the National Research Council (NRC, 2012). The zinc concentration in the basal diets was 150 mg/kg, provided in the form of ZnSO_4_. The piglets were housed on plastic slotted floors (1.8 m × 1.0 m per pen). The experiment lasted for 28 days. The temperature was kept around 26°C and humidity was constantly maintained at 55%. Water was provided to piglets *ad libitum*. The piglets were fed three times per day (8:00 am, 12:00 pm, and 17:00 pm) during the experiment.

### Sampling and DNA Extraction

At the end of the experiment (day 28), one piglet per replicate was randomly selected from four replicates per group (*n* = 4) for slaughter to harvest the intestinal samples. Intestinal contents were taken from the ileum and colon, shock-frozen in liquid nitrogen and immediately stored at -80°C for further experiments. Microbial genomic DNA was extracted from 200 mg of sample using QIAamp DNA stool minikit (Qiagen, Germany) according to the manufacturer’s instructions. Purified DNA isolation was confirmed by agarose gel electrophoresis and quantified using a ND-2000C spectrophotometer (NanoDrop, USA). Absorbance ratios at 260/280 nm and at 260/230 nm were determined to quantify and assess the purity of DNA samples.

### 16S rRNA Genes Amplification and Sequencing

2.5 microliter diluted DNA samples (5 ng/μL) were used for 25 μL PCR reaction mixtures. A pair of 10 μL primers with 1 μM concentrations of forward primer (341F 5′- CCTACGGGAGGCAGCAG-3′) and the reverse primer (806R 5′- GGACTACHVGGGTWTCTAAT-3′) with attaching 12 bp barcode sequences were used to amplify a region covering the V3-V4 region of bacterial 16S rRNA genes (Weisburg et al., 1991; Caporaso et al., 2011). The PCR conditions were as follows: one pre-denaturation cycle at 95°C for 3 min, denaturation by 25 cycles of 95°C for 30 s, annealing at 55°C for 30 s, elongation at 72°C for 30 s, and then a final extension at 72°C for 5 min and holding at 4°C. The PCR amplicon products were purified using agarose gel electrophoresis and were extracted to construct the sequencing library. The libraries of amplicons were attached to Illumina sequencing adapters using the NEBNext Ultra^TM^ II DNA Library Prep Kit for Illumina (E7645L), and then were cleaned up using AMPure XP beads (Biomek, USA) followed by quality control on an Agilent 2100 bioanalyzer (Agilent, USA). Then pooled libraries were pair-end sequenced on the Illumina MiSeq platform with using 2 × 250 bp MiSeq reagent kit v2 (Illumina, USA).

### Sequences Processing and Bioinformatics Analysis

In order to obtain high quality sequences, the head or tail bases whose qualities were lower than Q30 were trimmed, and sequence lengths shorter than 100 bp were removed. The fastq-join (v1.3.1) (Aronesty, 2013) was used to combine the paired-end reads. The combined sequences were further filtered because of ambiguous bases exceeding 6 bp, homopolymer runs exceeding 6 bp and primer mismatches exceeding 3 bp in QIIME (v1.8.0) (Caporaso et al., 2010). The filtered sequences were uploaded to QIIME to obtain OTU (Operational Taxonomic Units) that each clustered OTU had 97% similarity level by using the UCLUST method (uclust v1.2.22) (Edgar, 2010). Representative sequences of OTUs were selected according to the maximum length and were aligned to the Greengenes 16S rRNA gene database (v13.8) (McDonald et al., 2012) by using the RDP classifier (v.2.2) (Wang et al., 2007) to obtain the taxonomy assignment. Relative abundance (%) of individual taxonomy was estimated as a putative value of phylum, class, order, family, and genus. The data was proceeded to analyze the alpha and beta diversity. The alpha-diversity indices (Chao1, Shannon, PD and observed species) were calculated based on a subset of randomly selected sequences (5,000, 10,000, 15,000, 20,000, 25,000, and 30,000) from each sample. Beta-diversity of PCA (principal components analysis) and weighted UniFrac-based PCoA (principal coordinate analysis) were both calculated to show the group differences. The beta-diversity statistical analyses were tested using PERMANOVA (permutational multivariate analysis of variance) based on Bray–Curtis dissimilarities and 999 permutations in the vegan package (v.2.3.2) (Oksanen et al., 2015) in R software (v.3.2.0).

To obtain the strains level identification, the based alignment method of the BLAST (Basic Local Alignment Search Tool) algorithm (blast v2.2.25) was used to align all OTUs sequences to the bacterial genome sequences that had been reported in GenBank. Then the alignment results were parsed according to the following criteria: (1) best hits; (2) screening the cutoff up to 97% identity and >200 bp aligned length or up to 90% identity and >400 bp alignment.

### Microbial Functional Predictions and Statistical Analyses

Microbial functions were predicted by PICRUSt (Phylogenetic Investigation of Communities by Reconstruction of Unobserved States) (v1.1.0) (Langille et al., 2013). Based on the precalculated GreenGenes (v13.5) database (Langille et al., 2013), PICRUSt was performed on the abundance predictions of the KEGG (Kyoto Encyclopedia of Genes and Genomes) orthologs and KEGG pathways of bacterial communities. The functional differences among groups were compared through the software STAMP (Statistical Analysis of Metagenomic Profiles) (Parks et al., 2014). Two-sided Welch’s *t*-test and Benjamini–Hochberg FDR correction were used in two-group analysis and ANOVA with the Tukey-Kramer test and Benjamini–Hochberg correction were chosen for multiple-group analysis. Statistical analysis of the ileal and colonic taxonomy between two groups were conducted for normal distribution by student t-test in Excel. Differences were considered significant at *P* < 0.05. Heatmap diagrams and other plotting were carried out in the R environment (v3.1.2)^1^.

### Sequence Data Accession Number

Raw paired-end reads per sample without barcode and primer bases were submitted to the Sequence Read Archive of the NCBI (accession number: SRP095386).

## Results

### Characteristics of Bacterial Community Libraries

An average of 37,083 and 34,729 high quality sequences per sample were obtained in the ileal and colonic microbiota, respectively (Supplementary Table S2). Further, these sequences in the ileal and colonic contents were assigned to 5,251 and 7,395 OTUs (operational taxonomic units) based on a 97% sequence similarity and at least five reads of total samples supported per OTU (Supplementary Table S3).

### Effects of ZnO and Antibiotics on Bacterial Alpha- and Beta-Diversity of the Ileal and Colonic Microbiota

Alpha diversity was estimated by richness indices (observed species and Chao1) and diversity indices (PD whole tree and Shannon). In ileal samples, both microbial richness (revealed by Chao1) and diversity (PD whole tree) in the ZnO or antibiotics-treated group had significantly increased (*P* < 0.05), compared to the control (non-treated) (**Table 1** and Supplementary Figure S1). Instead, in colonic contents, microbial richness (observed species and Chao1) and diversity (PD whole tree) were significantly lower (*P* < 0.05) in both the ZnO- or antibiotics-treated group than that in control group (**Table 1** and Supplementary Figure S1). However, in both ileal and colonic contents, there was no significant difference between ZnO and antibiotics-treatments (*P* > 0.05), indicating they have sample bactericide effects (**Table 1** and Supplementary Figure S1).

**Table 1:** Richness and diversity indices estimation of intestinal microbiota (based on 13,800 sequences per sample).

Groups	Richness indices		Diversity indices	
	Observed species	Chao1	PD whole tree	Shannon
**ILEUM**				
Control (*n* = 4)	1713.50 ± 98.66	2905.81 ± 209.69	87.94 ± 3.27	7.26 ± 0.29
Antibiotics (*n* = 4)	1800.20 ± 140.37	3521.36 ± 258.69	100.65 ± 5.46	6.82 ± 0.44
zinc oxide (ZnO) (*n* = 4)	1733.30 ± 115.41	3277.46 ± 150.33	96.74 ± 6.51	6.83 ± 0.42
*P*.value (Antibiotics vs. Control)	0.42	**0.02**	**0.01**	0.19
*P*.value (ZnO vs. Control)	0.83	**0.03**	**0.04**	0.2
*P*.value (ZnO vs. Antibiotics)	0.55	0.21	0.46	0.96
**COLON**				
Control (*n* = 4)	2703.35 ± 75.76	4809.94 ± 371.91	137.94 ± 3.96	8.91 ± 0.21
Antibiotics (*n* = 4)	2452.23 ± 102.21	4337.07 ± 252.35	130.25 ± 4.76	8.69 ± 0.12
ZnO (*n* = 3)	2496.43 ± 28.61	4586.71 ± 241.31	131.94 ± 0.77	8.54 ± 0.19
*P*.value (Antibiotics vs Control)	**0.01**	**0.02**	**0.04**	0.16
*P*.value (ZnO vs. Control)	**0.01**	**0.04**	**0.01**	0.08
*P*.value (ZnO vs. Antibiotics)	0.56	0.28	0.63	0.25

*Significant *p*-values (< 0.05) are bolded.*

**FIGURE 1:**
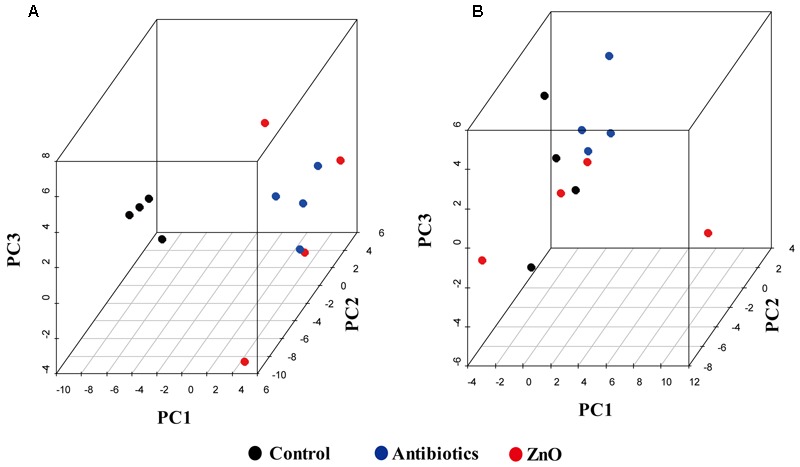
**Principal components analysis (PCA). (A)** Scatterplot of bacterial composition in the ileal samples (*n* = 4). **(B)** Scatterplot of bacterial composition in the colonic samples (*n* = 4).

The relationships between the community structures of the piglet microbiota were investigated by using the PCA and PCoA. The PCoA indicated that there was distinctly difference in distribution of microbiota at the ileal and colonic contents, but groups were not distinctly clustered separately along the principal coordinate (Supplementary Figure S2). However, the distribution of microbiota at either the ileum or colon within groups were distinctly clustered separately based on PCA (**Figure 1**). The relationships in gut microbiota between groups were calculated by using PERMANOVA based on Bray–Curtis distance and genus level, and the results showed that community composition was not differed among the three groups (*P* > 0.05, **Tables 2, 3**).

**Table 2:** Pseudo *F* table of PERMANOVA analysis based on Bray–Curtis dissimilarities (based on genus level).

Source of variance	Sum of squares	Degrees of freedom	Mean square	*F*	*R*^2^	*p*.adjust
Ileum.Groups	0.34	2.00	0.17	1.28	0.22	0.32
Colon.Groups	0.35	2.00	0.17	1.57	0.28	0.08

**Table 3:** Pseudo *F* table of pairwise comparisons of diet types using PERMANOVA (based on genus level and *n* = 4 per group expect *n* = 3 in colonic ZnO group).

Pairwise comparison	Source of variance	*F*	*R*^2^	*p*.value	*p*.adjust
Antibiotics vs. Control	Ileum.Groups	2.32	0.28	0.17	0.52
ZnO vs. Control	Ileum.Groups	1.36	0.18	0.23	0.69
ZnO vs. Antibiotics	Ileum.Groups	0.39	0.06	0.62	1.00
Antibiotics vs. Control	Colon.Groups	1.02	0.15	0.45	1.00
ZnO vs. Control	Colon.Groups	1.59	0.24	0.18	0.55
ZnO vs. Antibiotics	Colon.Groups	2.19	0.30	0.07	0.22

### Effects of ZnO Supplementation on the Composition of the Ileal and Colonic Microbiota

**FIGURE 2:**
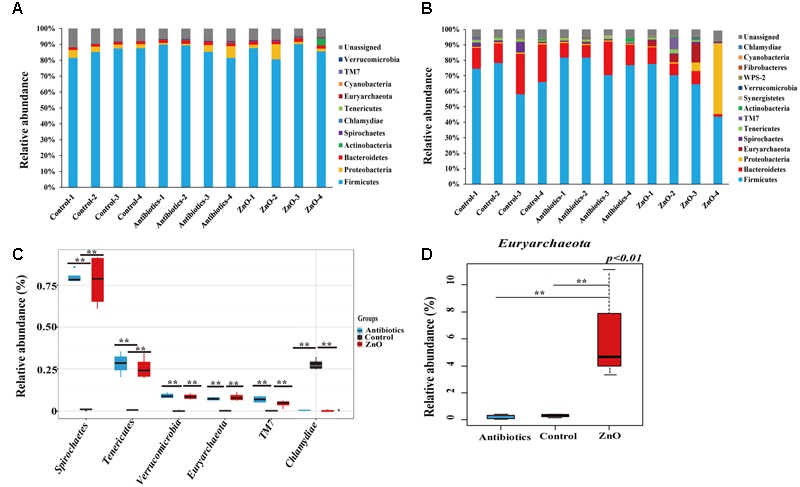
**Phyla distribution and comparison. (A)** Relative abundance (higher than 0.05%) of bacterial phyla in the ileum. *Firmicutes, Proteobacteria*, and *Bacteroidetes* were the predominant in all samples and *Firmicutes* comprised an average of 84% of the total sequences. **(B)** Relative abundance (higher than 0.05%) of bacterial phyla in the colon. *Firmicutes* and *Bacteroidetes* were the most predominant in all samples and *Firmicutes* comprised an average of 68% of the total sequences. The ratio of *Proteobacteria* reached to 43% in the sample of zinc oxide-4 (ZnO-4) which was excluded out any analysis. **(C)** The difference comparison at phylum level of ileal microbiota (*n* = 4). 5 phyla were significantly increased and 1 phyla were significantly reduced by high ZnO supplement or antibiotics supplement when compared with control. **(D)** The difference comparison at phylum level of colonic microbiota (*n* = 4 in control and antibiotics group, *n* = 3 in ZnO group). Only 1 phyla was significantly increased in the colon by high ZnO supplement when compared with antibiotics supplement or control. Statistics were conducted as a *t*-test between two groups; asterisk (^∗^) indicates *P* < 0.05 and double asterisk (^∗∗^) for *P* < 0.01.

After filtering the relative abundance of phyla lower than 0.05% in all groups, 11 and 13 phyla were identified in the ileal and colonic microbiota of weaned piglets, respectively (**Figures 2A,B** and Supplementary Tables S4, S5). *Firmicutes* (81∼90%), *Proteobacteria* (1-9%) and *Bacteroidetes* (1.7–2.4%) were the most predominant phyla in the ileal contents, together accounting for more than 91.5% of the total sequences (**Figure 2A** and Supplementary Table S4). In the colon, *Firmicutes* (72.80 ± 7.60%) and *Bacteroidetes* (14.14 ± 6.73%) were the most predominant phyla, occupying more than 94% of the total sequences (**Figure 2B** and Supplementary Table S5). A total of 7% and 4.8% of sequences could not be assigned to a phylum in the ileum and colon, respectively, indicating that there were some new bacteria members existing in the piglet. However, there were no significant difference detected in the most predominant phyla among the groups in either the ileum or colon (Supplementary Tables S4, S5), indicating that the ZnO and antibiotics supplementations did not change the predominant bacteria taxa at the phyla. However, when compared to the control, the proportion of *Spirochaetes, Tenericutes, Euryarchaeota, Verrucomicrobia*, and TM7 in the ileal digesta were significantly increased while its relative abundance of *Chlamydiae* were significantly decreased in the ZnO and antibiotics-supplemented groups (*P* < 0.05) (**Figure 2C**). In colonic microbiota, the relative abundance of *Euryarchaeota* in ZnO group was significantly higher than that in the control or antibiotics group (**Figure 2D**).

**FIGURE 3:**
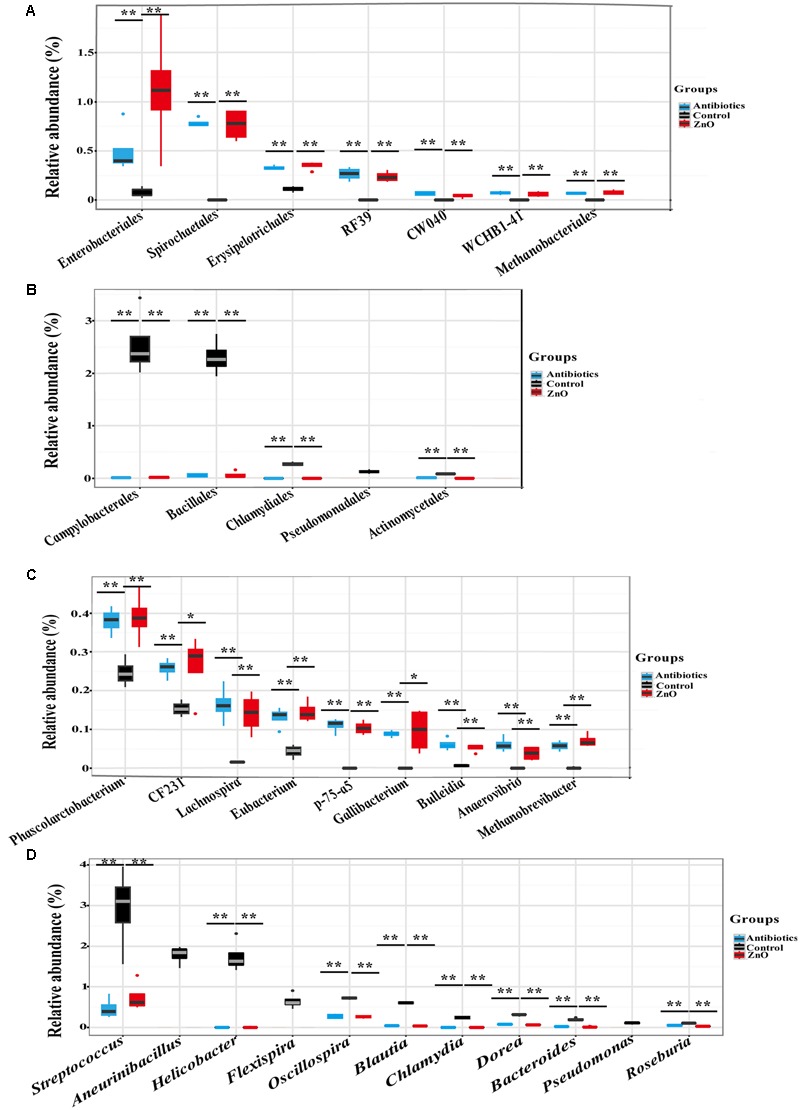
**The difference comparison of bacterial orders and genera between groups in the ileum. (A)** The significant increase of orders. Seven orders including *Enterobacteriales, Spirochaetales, RF39, Erysipelotrichales*, and so on were significantly increased by high zinc supplement or antibiotics supplement when compared with control. **(B)** The significant decrease of orders. Five orders including *Campylobacterales, Bacillales, Chlamydiales, Pseudomonadales*, and *Actinomycetales* were significantly reduced by high zinc supplement or antibiotics supplement when compared with control. **(C)** The significant increase of genera. Genera including *Phascolarctobacterium, CF231, Lachnospira, Eubacterium*, and so on were significantly increased by high zinc supplement or antibiotics supplement when compared with control. **(D)** The significant decrease of genera. Genera including *Helicobacter, Streptococcus, Aneurinibacillus, Flexispira*, and so on were significantly reduced by high zinc supplement or antibiotics supplement when compared with control. Statistics were conducted as a *t*-test between two groups (*n* = 4 per group); asterisk (^∗^) indicates *P* < 0.05 and double asterisk (^∗∗^) for *P* < 0.01.

In addition, after filtering the relative abundances lower than 0.05% in all groups, a total of 16 and 19 classes (Supplementary Tables S6, S7), 21 and 22 orders (Supplementary Tables S8, S9), 31 and 36 families (Supplementary Tables S10, S11), 43 and 43 genera (Supplementary Tables S12, S13) were identified in the ileal and colonic microbiota, respectively. High dietary ZnO level had a great effect on the order level of ileal microbiota. *Clostridiales* (39∼59%) and *Lactobacillales* (24∼44%) were found as the most predominant orders in the control, antibiotics, and ZnO groups, respectively. Antibiotics and ZnO supplementations both numerically increased the relative abundance of *Clostridiales* and numerically decreased the relative abundance of *Lactobacillales* (*P* > 0.05). Noteworthy changes in ileal microbiota in several bacterial orders were found in the ZnO group or antibiotics group. For example, the relative abundance of seven bacterial orders including *Enterobacteriales, Spirochaetales, Erysipelotrichales, RF39*, CW040, WCHB1-41, and *Methanobacteriales* in ZnO and antibiotics groups was significantly higher than that in the control (*P* < 0.05) (**Figure 3A**). It is worthy of highlighting that the relative abundance of *Enterobacteriales* was at least 33 and 6 higher in the ZnO and antibiotics group than the control (*P* < 0.05). Instead, the relative abundance of *Campylobacterales, Bacillales, Chlamydiales, Pseudomonadales*, and *Actinomycetales* decreased in ZnO or antibiotics-treated group when compared to the control (*P* < 0.05) (**Figure 3B**). Notably, the relative abundance of *Campylobacterales* in the ZnO and antibiotics groups showed 0.005- and 0.006-fold lower than that in the control (*P* < 0.05). Similarly, in colonic microbiota, *Clostridiales* (54∼68%), *Bacteroidales* (9∼19%) and *Lactobacillales* (3∼13%) were found as the most predominant orders in the control, antibiotics, and ZnO groups, respectively, but no significant differences were observed in these predominant orders among these treatments. However, high dietary ZnO significantly increased 21- and 30-fold of *Methanobacteriales* when compared with control or antibiotics group (*P* < 0.05) (Supplementary Figure S3). It is worthy of highlighting that antibiotics-treated group significantly increased the relative abundance of *Enterobacteriales* when compared with control or ZnO group (*P* < 0.05). Instead, ZnO-treated group numerically increased the relative abundance of *Enterobacteriales* when compared with control (Supplementary Figure S3).

On the genus level, *Lactobacillus* and *Clostridium* were the most predominant genera in the ileal digesta, while *Prevotella* and *Lactobacillus* were dominanted in the colonic digesta. Similarly, to the predominant phyla and orders, there were no significant differences in predominant genera between two groups. However, most non-domain genera of ileal microbiota were affected by ZnO and antibiotics treatments. The relative abundance of genera including *Phascolarctobacterium, CF231, Lachnospira, Eubacterium*, p-75-a5, *Gallibacterium, Bulleidia, Anaerovibrio*, and *Methanobrevibacter* were significantly increased in high dietary ZnO group or antibiotics group (*P* < 0.05) (**Figure 3C**). Under the same conditions, a significant decrease in relative abundance of genera, including *Streptococcus, Aneurinibacillus, Helicobacter, Flexispira, Oscillospira, Blautia, Chlamydia, Dorea, Bacteroides, Pseudomonas*, and *Roseburia* were found in ileum (*P* < 0.05) (**Figure 3D**). In the colonic contents, the relative abundance of *Methanobrevibacter* was significantly increased more than 20-fold in ZnO-treated group when compared with control or antibiotics group (*P* < 0.05) (Supplementary Figure S3).

**FIGURE 4:**
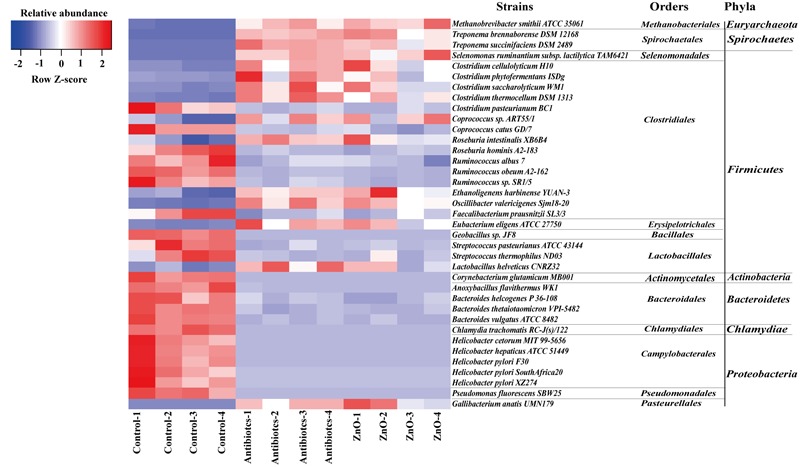
**The difference comparison of strains between groups in the ileum.** About 15 bacterial strains of ileal microbiota were both increased in ZnO group or antibiotics group, such as 4 *Clostridium* strains, 2 *Treponema* strains, 1 *Lactobacillus* strain, 1 *Eubacterium* strain, 1 *Methanobrevibacter* strain, 1 *Selenomonas* strain, and so on. Meanwhile, about 22 bacterial strains of ileal microbiota were both reduced in ZnO group or antibiotics group, such as 5 *Helicobacter* strains, 3 *Ruminococcus* strains, 3 *Bacteroides* strains, 2 *Streptococcus* strains, 1 *Chlamydia* strain, 1 *Pseudomonas* strain, and so on. Statistics were conducted by ANOVA with Tukey–Kramer test and Benjamini–Hochberg correction among three groups (*n* = 4), and the *P*-value lower than 0.05 and the relative abundance higher than 0.05% were shown.

We further assessed differences in the total bacterial community at strains level with adopting the blast algorithm of aligning the assembled 16s rRNA sequences to the genome of bacterial strains and parsed the alignment results with several filtered settings (see Materials and Methods). A total of 260 and 210 bacterial strains were identified in the ileal and colonic contents, respectively. The relative abundance (higher than 0.05%) of about 15 bacterial strains of ileal microbiota were both increased in ZnO group or antibiotics group, such as 4 *Clostridium* strains, 2 *Treponema* strains, 1 *Lactobacillus* strains, 1 *Eubacterium* strains, 1 *Methanobrevibacter* strain, 1 *Selenomonas* strain, and so on (**Figure 4** and Supplementary Table S14). Meanwhile, the relative abundance (higher than 0.05%) of about 22 bacterial strains in the ileal digesta were both reduced in ZnO group or antibiotics group, such as 5 *Helicobacter* strains, 3 *Ruminococcus* strains, 3 *Bacteroides* strains, 2 *Streptococcus* strains, 1 *Chlamydia* strains, 1 *Pseudomonas* strains, and so on (**Figure 4** and Supplementary Table S14). In the colonic digesta, the relative abundance of *Methanobrevibacter smithii* strain was significantly increased 20-fold in the ZnO group when compared with control and antibiotics group (Supplementary Table S15).

### Microbiota Function Prediction

**FIGURE 5:**
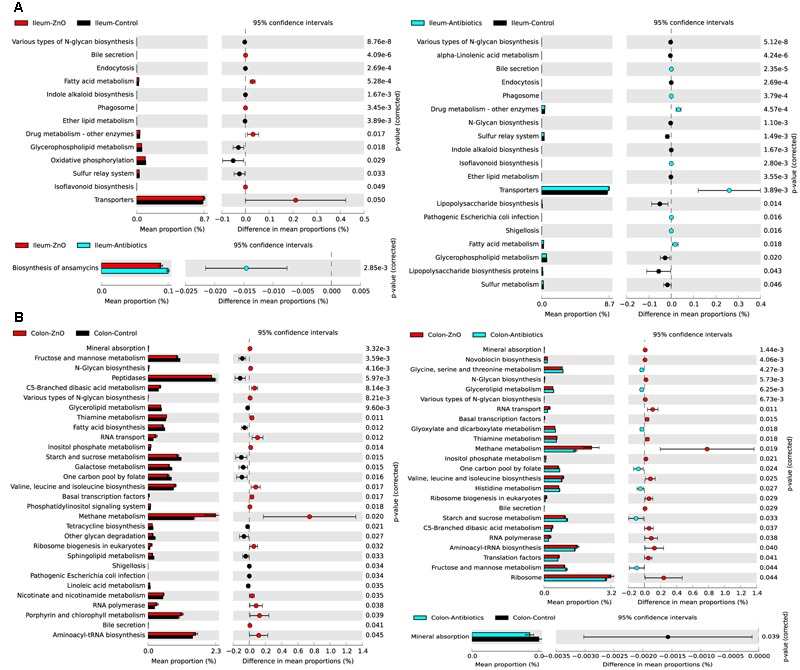
**Predicted microbial function comparison. (A)** Comparing ileal microbial function between zinc oxide (ZnO) (*n* = 4), antibiotics (*n* = 4), and control (*n* = 4) groups. High zinc or antibiotics supplement both significantly increased the function enrichment of transporters, fatty acid metabolism, and drug metabolism. **(B)** Comparing colonic microbial function between ZnO (*n* = 3), antibiotics (*n* = 4), and control (*n* = 4). High zinc supplement significantly increased the function enrichment of methane metabolism when compared with control or antibiotics. Statistics were conducted by two-sided Welch’s *t*-test and Benjamini–Hochberg FDR correction between two groups, and the *P*-value of different functions lower than 0.05 were shown.

In order to investigate the functional profiles among three groups, we used a computational tool, PICRUSt. The ileal microbiota in the ZnO group or antibiotics group both had higher enrichments of functions involved in transporters, fatty acid and drug metabolism (**Figure 5A**). The abundance of predicted microbial functions was almost no statistically difference between ZnO group and antibiotics group in ileum. For the colonic digesta, the ZnO group had higher abundance of microbiota functions involved in energy and methane metabolism, and lower abundance of functions in starch, sucrose, fructose as well as mannose metabolism (**Figure 5B**).

**FIGURE 6:**
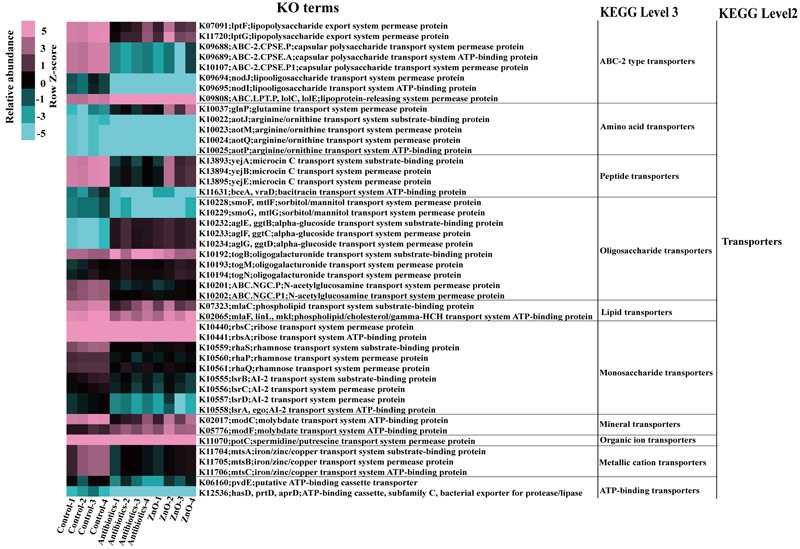
**Transporters pathway.** The abundant of KO terms that enriched on transporters pathway of ileal microbiota, including ABC-2 type transporters, amino acid transporters, peptide transporters, oligosaccharide transporters, lipid transporters, and so on, were significantly influenced by high zinc or antibiotics supplement. Statistics were conducted by ANOVA with Tukey–Kramer test and Benjamini–Hochberg correction among three groups (*n* = 4), and the *P*-value lower than 0.05 and the relative abundance higher than 0.01% were shown.

We analyzed transporters pathway of the ileal microbiota using the KO terms. Significant differences were observed in ABC-2 type transporters, amino acid transporters, and oligosaccharide transporters (**Figure 6**). The proportions of ABC-2 type transporters, lipopolysaccharide export system permease protein (lptF, lptG), lipooligosaccharide (LOS) transport system permease protein (nodJ) ATP-binding protein (nodI), capsular polysaccharide transport system permease protein (E.P), and ATP-binding protein (CPSE.A), were significantly decreased in the ZnO group or antibiotics group (*P* < 0.05). Interestingly, the abundance of lipoprotein-releasing system permease proteins (ABC.LPT.P) was significantly increased in the ZnO group or antibiotics group (*P* < 0.05). Within amino acid transporters, the abundance of glutamine transport system permease protein (glnP) was greatly increased sixfold (log2) in ZnO-treated group and threefold (log2) in antibiotics group (*P* < 0.05). In regards to oligosaccharide transporters, the abundance of alpha-glucoside transport system permease protein (aglF) and substrate-binding protein (aglE) was increased sixfold (log2) in the ZnO group or antibiotics group (*P* < 0.05).

The KO terms enriched on methane metabolism pathway of colonic microbiota were further analyzed (Supplementary Figure S4). Most of methane metabolism related KO terms, whose corresponding OTUs belonging to *Methanobrevibacter* genus, were significantly increased by high dietary ZnO supplementation. The methyl-coenzyme M reductase and heterodisulfide reductase, participating in the key enzymes related to methane production, ATP synthesis and energy storage, were significantly increased in the ZnO group when compared with the control group or antibiotics group. Coenzyme M (CoM) was very important in methane metabolism. The abundance of 2-phosphosulfolactate phosphatase (comB), sulfopyruvate decarboxylase subunit alpha (comD), and subunit beta (comE) that involved in coenzyme M biosynthesis were remarkably increased in the ZnO group when compared with the other two groups. These results implied that ZnO possibly stimulated the methane production and energy supply.

## Discussion

In the present study, we provided an in-depth analysis of the bacterial taxa compositions and compared the effects of the ZnO and antibiotics supplements on microbial community structures in the ileum and colon of weaned piglets. Our results showed the piglets fed the diets supplemented with high ZnO had similar microbiome profiles in the ileal and colonic digesta compared to those in the antibiotics group.

Our discoveries here showed that both high dietary ZnO and in-feed antibiotics significantly increased the microbiota diversity of the ileal digesta while they decreased the microbiota diversity of the colonic digesta (**Table 1**). The antibiotic colistin sulfate has been shown to increase the diversity of the cecum microbiota in weaned pigs (Ye et al., 2015). Starke et al. (2014) showed that the total bacterial diversity was significantly increased in the small intestinal microbiota of piglets which were fed the high zinc diet. On the other hand, another study showed that that antibiotics decreased the microbial richness and diversity of fecal bacterial communities, and decreased the incidence of diarrhea of piglets (Wang et al., 2012). Furthermore, pharmacological doses of ZnO have been shown to have an effect on regulation of the microbial compositions by reducing the microbial richness in the jejunum and feces (Shen et al., 2014), or the ileal digesta (Namkung et al., 2006) of weaned piglets. Accordingly, our results showed that the bacterial richness was significantly higher in the ileal digesta while it was significantly lower in the colon digesta than that in control, due to the dietary supplement of ZnO or antibiotics (**Table 1**).

ZnO has a low solubility in the large intestine due to high pH, which generally leads to no effect on colonic microbiota (Pieper et al., 2012; Starke et al., 2014). Remarkably, we observed that the relative abundance of *Euryarchaeota* in the ileal and colonic contents were both increased by high dietary ZnO (up to 20-fold) compared to the control. *Euryarchaeota*, a phylum of the Archaea, includes some methanogens which are often found in intestines (Horz and Conrads, 2010). *Methanobrevibacter smithii* is the predominant archaeon in the human gut, playing an important role in the efficient digestion of polysaccharides by consuming the end products of bacterial fermentation; meanwhile, it is a methanogen converting the hydrogen and carbon dioxide into methane with the yield of energy (Armougom et al., 2009). The relative abundance of *Spirochaetes* was increased by high dietary ZnO or antibiotics. A previous study has identified a positive correlation between swine weight and the abundance of the family *Spirochaetaceae* (Unno et al., 2015). Similarly, the increased proportion of *Spirochaetaceae* by dietary high ZnO supplementation observed in this study may be linked to our early observation that dietary high ZnO improved BW of weaned piglets (Zhu et al., 2017). Furthermore, the percentages of *Verrucomicrobia* were significant higher in ZnO group or antibiotics group in the ileum. This is in agreement with the previous reports that broad-spectrum antibiotic treatment increased the high-level colonization of *Verrucomicrobia* in the human gut (Dubourg et al., 2013). Chlamydiae, were obligate intracellular pathogens (Wyrick, 2000) and susceptible to antibiotic treatment (Gupta, 2011). We observed ZnO reduced the population of *Chlamydiae* phylum, *Chlamydiales* order, *Chlamydia* genus, and *Chlamydia trachomatis* showing that ZnO can repress *Chlamydiae* as an antimicrobial agent.

On the order level, high ZnO and antibiotics dramatically increased the relative abundances of *Enterobacteriales*, while significantly decreased *Campylobacterales* and *Pseudomonadales*, all belonging to gram-negative phylum *Proteobacteria*. Many previous studies showed that there was a trend with an increase in *Enterobacteriales* in the ileum when high dietary ZnO was fed to piglets (Hojberg et al., 2005; Vahjen et al., 2010, 2011; Starke et al., 2014). Particularly, the in-feed antibiotics ASP250 (chlortetracycline, sulfamethazine and penicillin) supplementation affected the microbiome structure at different gut locations, with notable increases in *E. coli* populations in the ileum (Looft et al., 2014). The increased diversity of the *Enterobacteriales* may act beneficial and promote the competition for diarrhoegenic strains of coliforms during the first week after weaning to combat *E. coli* induced diarrhea in piglets (Katouli et al., 1999; Starke et al., 2014). In this study, we observed ZnO numberically increased non-pathogenic *E. coli, such as E. coli K-12*, maybe it cause the colonization competition of coliforms. Some *Campylobacterales* were opportunistic pathogens causing a life-threatening gastrointestinal disease (Xie et al., 2011). For example, *Helicobacter pylori* within the order *Campylobacterales* infects up to 50% of the human population and strongly associates with peptic ulcers, chronic gastritis, duodenitis, and stomach cancer (Yamaoka, 2008). It has been reported that *Campylobacter jejuni* was extremely sensitive to ZnO nanoparticles and could not be recovery (Xie et al., 2011). Similarly, we showed that high ZnO and antibiotics can effectively reduce the counts of *Helicobacter* genus, such as *Helicobacter pylori*. That means *Helicobacters* are not only susceptible to antibiotics, but also susceptible to high dietary ZnO supplementation.

With respect to metabolic pathways, the enrichment of “methane metabolism pathways” was remarkably increased in colon. For example, two key enzymes, methyl-coenzyme M reductase (Mcr) and heterodisulfide reductase (Hdr), involved in methane production, and ATP synthesis (Samuel et al., 2007), were significantly increased by ZnO addition. Both Mcrand Hdr enzymes require zinc ion as the cofactors (Tallant et al., 2001; Hamann et al., 2007), which may be provided by dietary ZnO; thus, ZnO probably confers to the activity of zinc-contained enzymes. Further study is warranted to elucidate the related mechanism.

“The transporter pathway” including lipopolysaccharide (LPS) export system permease protein, LOS transport system permease protein, and capsular polysaccharide transport system permease protein were significantly decreased in ZnO or antibiotics group. ABC transporter complex lptBFG is involved in the translocation of LPS from the inner membrane to the outer membrane (Narita and Tokuda, 2009). LOS are glycolipids found in the outer membrane of the Gram-negative bacteria. LOS, together with LPS, plays a central role in maintaining the integrity and functionality of the outer membrane of cell envelope (Moran et al., 1996). ZnO or antibiotics may reduce the exportation of LPS and LOS, and thereby lead to the outer membrane without structural integrity. For example, ZnO nanoparticles disrupted the cell morphology, membrane integrity, and cause oxidative stress in *Campylobacter* (Xie et al., 2011). Capsular polysaccharides as virulence factor contributes adherence of pathogens to epithelial surfaces (Lesinski and Westerink, 2001). Dietary high ZnO reduced the incidence of LPS-induced bacterial translocation from the small intestine of young pigs (Huang et al., 1999). Interestingly, the abundance of glutamine transport system permease protein and alpha-glucoside transport system permease protein were significantly increased in high dietary ZnO and antibiotics groups. Previous studies showed that glutamine transport system permease protein mutants were unable to transport L-glutamine, indicating their important roles in the L-glutamine uptake system (Masters and Hong, 1981; Nohno et al., 1986; Pistolesi and Tjandra, 2012). The increase of glutamine transport system permease protein could be due to high abundance of *E. coli* strains, which carry a high affinity, binding protein dependent L-glutamine uptake system with glnH, glnP, and glnQ encode proteins (Berger et al., 1974). Higher abundant of glnP due to supplements of ZnO or antibiotics may increase the absorption of L-glutamine and provide the nitrogen source to *E. coli*.

In summary, dietary high ZnO modulated the gut microbial diversity and altered the microbial community in the ileum and colon of weaned piglets, and showed comparable effects to in-feed antibiotics, especially affected the population of non-predominant microbiota in the ileal digesta. The understanding on effects of high ZnO on intestinal bacterial communities may provide insights into future application of the alternative strategy for treating diarrhea in piglets.

## Author Contributions

The authors’ contributions are as follows: ZJ and ZC designed the research. HL, RF, QZ, and JX conducted the research. TY and LG analyzed the data. SC, CZ, and TY wrote the manuscript. All authors read and approved the final version of the manuscript.

## Conflict of Interest Statement

The authors declare that the research was conducted in the absence of any commercial or financial relationships that could be construed as a potential conflict of interest.

## Footnotes

1 http://www.r-project.org
