# Supplementary material for: Dietary High Zinc Oxide Modulates the Microbiome of Ileum and Colon in Weaned Piglets
Source: Front Microbiol. 2017 May 9;8:825. doi: 10.3389/fmicb.2017.00825 (PMC5422713; doi:10.3389/fmicb.2017.00825)
Supplement: Supplementary file 1 [file Data_Sheet_1.docx]

**Table S1. Ingredient and composition of the basal diet (as fed basis).**

| **Ingredients** | **%** | **Calculated nutrient level** |  |
| --- | --- | --- | --- |
| Corn | 60.43 | DE, MJ/kg | 14.10 |
| Extruded soybean meal | 20.00 | CP, % | 19.20 |
| Fish meal | 5.00 | Lys, % | 1.17 |
| Soy protein concentrate | 2.00 | Met, % | 0.50 |
| Whey powder (3.6% CP) | 7.50 | Met + Cys, % | 0.74 |
| Soybean oil | 1.50 | Ca, % | 0.85 |
| CaHPO_4_ | 1.05 | Total P, % | 0.68 |
| Salt | 0.30 | Available P, % | 0.49 |
| Limestone | 0.75 |  |  |
| L-Lys-HCl | 0.25 |  |  |
| DL-Met | 0.10 |  |  |
| Choline chloride (50%) | 0.12 |  |  |
| Vitamin and mineral Premix^1^ | 1.00 |  |  |
| Total | 100.00 |  |  |

^1^Supplied per kilogram of diet: vitamin A, 11,000 IU; vitamin D_3_, 1,100 IU; vitamin E, 80 mg; vitamin K_3_,2.5 mg; vitamin B_12_, 100 μg; thiamin, 7.5 mg; riboflavin, 20 mg; niacin, 100 mg; pantothenic acid, 60 mg; folic acid, 1.5 mg; pyridoxine, 10 mg; biotin 0.4 mg; choline chloride, 500 mg; Fe, 150 mg; Cu, 10 mg; Mn, 10 mg; Zn, 150 mg; I, 0.2 mg; Se, 0.3 mg; Co, 0.15 mg.

**Table S2. The sequences of each sample in ileal and colonic contents.**

| **Samples** | **Ileal contents** | **Colonic contents** |
| --- | --- | --- |
| Control-1 | 68,275 | 32,025 |
| Control-2 | 66,595 | 37,422 |
| Control-3 | 59,093 | 48,529 |
| Control-4 | 43,255 | 40,890 |
| Antibiotics-1 | 28,980 | 33,859 |
| Antibiotics-2 | 26,906 | 33,879 |
| Antibiotics-3 | 26,300 | 32,867 |
| Antibiotics-4 | 37,378 | 29,332 |
| ZnO-1 | 16,971 | 39,107 |
| ZnO-2 | 20,018 | 36,442 |
| ZnO-3 | 18,138 | 25,983 |
| ZnO-4 | 33,086 | 26,407 |
| **Summary** |  |  |
| Num samples | 12 | 12 |
| Total Reads | 444,995 | 416,742 |
| Min reads/sample | 16,971 | 25,983 |
| Max reads/sample | 68,275 | 48,529 |
| Mean reads/sample | 37,083 | 34,729 |

**Table S3. The OTU numbers of each group in ileal and colonic contents (n=4 per group).**

| **Groups** | **Ileal contents** | **Colonic contents** |
| --- | --- | --- |
| Control group | 3061 | 3291 |
| Antibiotic group | 1655 | 2588 |
| ZnO group | 1343 | 2337 |
| Total OTU in all groups | 5251 | 7395 |
| Common OTU in all groups | 534 (10%) | 1113 (15%) |

The OTUs were identified based on 97% sequence similarity and at least five reads of total samples supported per OTU.

**Table S4.The phyla composition of ileal microbiome in each group (n=4 per group).**

| **Phyla** | **Control**  **group** | **Antibiotics group** | **ZnO**  **group** | **P.value**  **(Antibiotics VS Control)** | **P.value**  **(ZnO VS Control)** | **P.value**  **(ZnO VS Antibiotics)** |
| --- | --- | --- | --- | --- | --- | --- |
|  |  |  |  |  |  |  |
| Firmicutes | 85.45±2.82% | 86.51±3.95% | 86.04±4.01% | 0.68 | 0.82 | 0.87 |
| Proteobacteria | 3.35±1.14% | 3.42±3.08% | 3.68±3.83% | 0.97 | 0.88 | 0.92 |
| Bacteroidetes | 1.76±0.07% | 2.05±0.15% | 2.10±0.23% | 0.25 | 0.27 | 0.74 |
| **Chlamydiae** | 0.28±0.03% | 0.00±0.00% | 0.00±0.00% | **0.00** | **0.00** | 0.18 |
| Actinobacteria | 0.11±0.01% | 0.19±0.15% | 1.29±2.06% | 0.33 | 0.29 | 0.33 |
| Cyanobacteria | 0.06±0.04% | 0.05±0.02% | 0.05±0.02% | 0.74 | 0.75 | 0.97 |
| **Spirochaetes** | 0.01±0.00% | 0.77±0.04% | 0.75±0.16% | **0.00** | **0.00** | 0.81 |
| **Tenericutes** | 0.00±0.00% | 0.24±0.06% | 0.21±0.06% | **0.00** | **0.00** | 0.45 |
| **Euryarchaeota** | 0.00±0.00% | 0.08±0.01% | 0.09±0.02% | **0.00** | **0.00** | 0.58 |
| **Verrucomicrobia** | 0.00±0.00% | 0.08±0.01% | 0.07±0.01% | **0.00** | **0.00** | 0.18 |
| **TM7** | 0.00±0.00% | 0.07±0.02% | 0.05±0.02% | **0.00** | **0.01** | 0.14 |
| **Unassigned** | 8.95±1.74% | 6.48±0.72% | 5.65±1.03% | **0.04** | **0.02** | 0.23 |

The relative abundance of phyla higher than 0.05% in any group were shown. The predominant phyla (the average abundance of all samples higher than 1%) including *Firmicutes,Proteobacteria* and *Bacteroidetes* showed no significant differences between two groups.The proportion of *Spirochaetes, Tenericutes, Euryarchaeota*, *Verrucomicrobia* and *TM7* in both ZnO and antibiotics-treated ileal samples were significantly higher than that in the control group (*P* < 0.05). Instead, the relative abundance of *Chlamydiae* in both ZnO and antibiotics-treated ileal samples were significantly lower than that in the control group (*P* < 0.05).

**Table S5.The phyla composition of colonic microbiome in each group.**

| **Phyla** | **Control group**  **(n=4)** | **Antibiotics group**  **(n=4)** | **ZnO**  **group**  **(n=3)** | **P.value**  **(Antibiotics VS Control)** | **P.value**  **(ZnO VS Control)** | **P.value**  **(ZnO VS Antibiotics)** |
| --- | --- | --- | --- | --- | --- | --- |
|  |  |  |  |  |  |  |
| Firmicutes | 69.28±9.03% | 77.77±5.40% | 70.87±6.54% | 0.16 | 0.81 | 0.19 |
| Bacteroidetes | 19.10±7.06% | 13.06±6.16% | 8.96±1.68% | 0.24 | 0.06 | 0.32 |
| Spirochaetes | 2.66±2.84% | 0.71±0.12% | 0.84±0.21% | 0.22 | 0.33 | 0.37 |
| Tenericutes | 1.41±0.47% | 1.31±0.50% | 1.66±0.81% | 0.78 | 0.63 | 0.51 |
| Proteobacteria | 0.77±0.07% | 0.72±0.06% | 2.32±2.76% | 0.28 | 0.30 | 0.28 |
| TM7 | 0.48±0.69% | 0.26±0.23% | 2.86±3.94% | 0.57 | 0.28 | 0.23 |
| **Euryarchaeota** | 0.35±0.12% | 0.24±0.18% | 7.03±4.60% | 0.37 | **0.03** | **0.03** |
| Actinobacteria | 0.33±0.39% | 0.67±1.10% | 0.49±0.25% | 0.59 | 0.58 | 0.80 |
| Cyanobacteria | 0.17±0.12% | 0.07±0.03% | 0.18±0.13% | 0.17 | 0.98 | 0.18 |
| Verrucomicrobia | 0.07±0.02% | 0.10±0.06% | 0.21±0.20% | 0.36 | 0.21 | 0.33 |
| Fibrobacteres | 0.04±0.03% | 0.11±0.19% | 0.02±0.01% | 0.48 | 0.50 | 0.48 |
| Synergistetes | 0.01±0.01% | 0.23±0.42% | 0.03±0.03% | 0.34 | 0.27 | 0.46 |
| WPS-2 | 0.00±0.00% | 0.01±0.01% | 0.20±0.20% | 0.24 | 0.11 | 0.12 |
| Unassigned | 5.30±0.47% | 4.71±0.71% | 4.33±0.24% | 0.21 | **0.02** | 0.41 |

The relative abundance of phyla higher than 0.05% in any group were shown. The most predominant phyla (the average abundance of all samples higher than 10%) including *Firmicutes* and *Bacteroidetes* showed no significant differences between two groups. The relative abundance of *Euryarchaeota* in ZnO group was significantly higher than that in the control or antibiotics group in colonic microbiota (*P* < 0.05).

**Table S6.The classes composition of ileal microbiome in each group (n=4 per group).**

| **Classes** | **Control**  **group** | **Antibiotics group** | **ZnO**  **group** | **P.value**  **(Antibiotics VS Control)** | **P.value**  **(ZnO VS Control)** | **P.value**  **(ZnO VS Antibiotics)** |
| --- | --- | --- | --- | --- | --- | --- |
|  |  |  |  |  |  |  |
| Bacilli | 46.82±24.32% | 27.46±20.74% | 42.99±29.03% | 0.27 | 0.85 | 0.42 |
| Clostridia | 38.51±21.55% | 58.72±21.08% | 42.68±27.64% | 0.23 | 0.82 | 0.39 |
| **Epsilonproteobacteria** | 2.62±0.64% | 0.01±0.01% | 0.02±0.01% | **0** | **0** | 0.29 |
| Bacteroidia | 1.75±0.07% | 2.05±0.15% | 2.10±0.23% | 0.25 | 0.27 | 0.74 |
| Gammaproteobacteria | 0.69±0.51% | 3.32±3.08% | 3.58±3.82% | 0.14 | 0.18 | 0.92 |
| **Chlamydiia** | 0.28±0.03% | 0.00±0.00% | 0.00±0.00% | **0** | **0** | 0.18 |
| **Erysipelotrichi** | 0.10±0.02% | 0.32±0.03% | 0.34±0.04% | **0** | **0** | 0.73 |
| Actinobacteria | 0.08±0.01% | 0.14±0.12% | 1.23±2.04% | 0.41 | 0.3 | 0.33 |
| Chloroplast | 0.06±0.04% | 0.03±0.02% | 0.03±0.02% | 0.22 | 0.29 | 0.8 |
| Betaproteobacteria | 0.03±0.01% | 0.06±0.02% | 0.06±0.04% | 0.04 | 0.13 | 0.8 |
| Coriobacteriia | 0.03±0.01% | 0.05±0.03% | 0.06±0.04% | 0.14 | 0.16 | 0.66 |
| **Spirochaetes** | 0.01±0.00% | 0.77±0.04% | 0.75±0.16% | **0** | **0** | 0.81 |
| **Mollicutes** | 0.00±0.00% | 0.24±0.06% | 0.21±0.06% | **0** | **0** | 0.45 |
| **Methanobacteria** | 0.00±0.00% | 0.08±0.01% | 0.08±0.02% | **0** | **0** | 0.49 |
| **TM7-3** | 0.00±0.00% | 0.07±0.02% | 0.05±0.02% | **0** | **0.01** | 0.14 |
| **Verruco-5** | 0.00±0.00% | 0.06±0.02% | 0.05±0.02% | **0** | **0** | 0.18 |
| **Unassigned** | 8.95±1.74% | 6.48±0.72% | 5.65±1.03% | **0.04** | **0.02** | 0.23 |

The relative abundance of classes higher than 0.05% in any group were shown. The most predominant classes (the average abundance of all samples higher than 10%) including *Bacilli* and *Clostridia* showed no significant differences between two groups. The proportion of *Erysipelotrichi, Spirochaetes, Mollicutes*, *Methanobacteria,TM7-3* and *Verruco-5* in both ZnO and antibiotics-treated ileal samples were significantly higher than that in the control group (*P* < 0.05). Instead, the relative abundance of *Epsilonproteobacteria* and *Chlamydiia* in both ZnO and antibiotics-treated ileal samples were significantly lower than that in the control group (*P* < 0.05).

**Table S7.The classes composition of colonic microbiome in each group.**

| **Classes** | **Control group**  **(n=4)** | **Antibiotics group**  **(n=4)** | **ZnO**  **group**  **(n=3)** | **P.value**  **(Antibiotics VS Control)** | **P.value**  **(ZnO VS Control)** | **P.value**  **(ZnO VS Antibiotics)** |
| --- | --- | --- | --- | --- | --- | --- |
|  |  |  |  |  |  |  |
| Clostridia | 59.08±6.06% | 68.50±7.79% | 53.72±14.51% | 0.1 | 0.53 | 0.14 |
| Bacteroidia | 19.10±7.06% | 13.06±6.16% | 8.96±1.68% | 0.24 | 0.06 | 0.32 |
| Bacilli | 8.19±6.20% | 5.96±2.43% | 14.17±9.36% | 0.53 | 0.35 | 0.14 |
| Spirochaetes | 2.66±2.84% | 0.71±0.12% | 0.84±0.21% | 0.22 | 0.33 | 0.37 |
| Erysipelotrichi | 1.99±1.43% | 3.30±3.49% | 2.96±2.25% | 0.52 | 0.52 | 0.89 |
| Mollicutes | 1.41±0.47% | 1.31±0.50% | 1.66±0.81% | 0.78 | 0.63 | 0.51 |
| **Gammaproteobacteria** | 0.54±0.02% | 0.60±0.06% | 0.48±0.05% | 0.09 | 0.08 | **0.03** |
| TM7-3 | 0.48±0.69% | 0.26±0.23% | 2.86±3.94% | 0.57 | 0.28 | 0.23 |
| **Methanobacteria** | 0.33±0.13% | 0.24±0.18% | 7.02±4.60% | 0.41 | **0.03** | **0.03** |
| Coriobacteriia | 0.20±0.22% | 0.62±1.08% | 0.27±0.16% | 0.48 | 0.67 | 0.61 |
| 4C0d-2 | 0.17±0.12% | 0.07±0.03% | 0.17±0.13% | 0.16 | 0.97 | 0.19 |
| Actinobacteria | 0.13±0.18% | 0.05±0.02% | 0.22±0.18% | 0.4 | 0.55 | 0.12 |
| Epsilonproteobacteria | 0.11±0.08% | 0.03±0.00% | 0.19±0.21% | 0.07 | 0.51 | 0.17 |
| Betaproteobacteria | 0.06±0.03% | 0.05±0.01% | 1.49±2.41% | 0.8 | 0.27 | 0.27 |
| Deltaproteobacteria | 0.06±0.05% | 0.03±0.01% | 0.16±0.19% | 0.24 | 0.37 | 0.22 |
| Verruco-5 | 0.06±0.02% | 0.06±0.02% | 0.05±0.02% | 0.85 | 0.5 | 0.46 |
| Fibrobacteria | 0.04±0.03% | 0.11±0.19% | 0.02±0.01% | 0.48 | 0.5 | 0.48 |
| Synergistia | 0.01±0.01% | 0.23±0.42% | 0.03±0.03% | 0.34 | 0.27 | 0.46 |
| Verrucomicrobiae | 0.01±0.01% | 0.04±0.04% | 0.16±0.19% | 0.27 | 0.17 | 0.25 |
| Unassigned | 5.30±0.47% | 4.71±0.71% | 4.33±0.24% | 0.21 | 0.02 | 0.41 |

The relative abundance of classes higher than 0.05% in any group were shown. The most predominant classes (the average abundance of all samples higher than 10%) including *Clostridia* and *Bacteroidia* showed no significant differences between two groups. The relative abundance of *Methanobacteria* in ZnO group was significantly higher than that in the control or antibiotics group in colonic microbiota (*P* < 0.05).

**Table S8.The orders composition of ileal microbiome in each group (n=4 per group).**

| **Orders** | **Control**  **group** | **Antibiotics group** | **ZnO**  **group** | **P.value**  **(Antibiotics VS Control)** | **P.value**  **(ZnO VS Control)** | **P.value**  **(ZnO VS Antibiotics)** |
| --- | --- | --- | --- | --- | --- | --- |
|  |  |  |  |  |  |  |
| Lactobacillales | 43.95±24.53% | 24.31±22.21% | 39.90±31.36% | 0.28 | 0.85 | 0.45 |
| Clostridiales | 38.50±21.55% | 58.70±21.07% | 42.68±27.64% | 0.23 | 0.82 | 0.39 |
| **Campylobacterales** | 2.62±0.64% | 0.01±0.01% | 0.02±0.01% | **0** | **0** | 0.73 |
| **Bacillales** | 2.34±0.33% | 0.05±0.03% | 0.06±0.07% | **0** | **0** | 0.84 |
| Bacteroidales | 1.75±0.07% | 2.05±0.15% | 2.10±0.23% | 0.25 | 0.27 | 0.74 |
| Turicibacterales | 0.51±0.48% | 3.10±2.77% | 3.03±2.98% | 0.11 | 0.15 | 0.97 |
| Pasteurellales | 0.51±0.48% | 2.78±2.87% | 0.84±0.67% | 0.17 | 0.45 | 0.24 |
| **Chlamydiales** | 0.28±0.03% | 0.00±0.00% | 0.00±0.00% | **0** | **0** | 0.18 |
| **Erysipelotrichales** | 0.10±0.02% | 0.32±0.03% | 0.34±0.04% | **0** | **0** | 0.29 |
| **Pseudomonadales** | 0.10±0.02% | 0.00±0.00% | 0.00±0.00% | **0** | **0** | 0.36 |
| **Enterobacteriales** | 0.07±0.05% | 0.50±0.23% | 2.69±3.64% | **0.01** | **0.01** | 0.27 |
| **Actinomycetales** | 0.07±0.01% | 0.01±0.01% | 0.00±0.01% | **0** | **0** | 0.33 |
| Streptophyta | 0.06±0.04% | 0.03±0.02% | 0.03±0.02% | 0.22 | 0.29 | 0.8 |
| Coriobacteriales | 0.03±0.01% | 0.05±0.03% | 0.06±0.04% | 0.14 | 0.16 | 0.66 |
| Tremblayales | 0.03±0.01% | 0.05±0.02% | 0.05±0.04% | 0.05 | 0.31 | 0.8 |
| Bifidobacteriales | 0.01±0.00% | 0.13±0.13% | 1.22±2.04% | 0.13 | 0.28 | 0.32 |
| **Spirochaetales** | 0.00±0.00% | 0.76±0.04% | 0.74±0.16% | **0** | **0** | 0.84 |
| **RF39** | 0.00±0.00% | 0.23±0.06% | 0.19±0.04% | **0** | **0** | 0.3 |
| **Methanobacteriales** | 0.00±0.00% | 0.08±0.01% | 0.08±0.02% | **0** | **0** | 0.49 |
| **CW040** | 0.00±0.00% | 0.07±0.02% | 0.05±0.02% | **0** | **0.01** | 0.14 |
| **WCHB1-41** | 0.00±0.00% | 0.06±0.02% | 0.05±0.02% | **0** | **0** | 0.18 |
| **Unassigned** | 8.95±1.74% | 6.48±0.72% | 5.65±1.03% | **0.04** | **0.02** | 0.23 |

The relative abundance of orders higher than 0.05% in any group were shown. The most predominant orders (the average abundance of all samples higher than 10%) including *Lactobacillales* and *Clostridiales* showed no significant differences between two groups. The proportion of *Erysipelotrichales,Enterobacteriales, Spirochaetales, RF39,Methanobacteriales, CW040*and *WCHB1-41* in both ZnO and antibiotics-treated ileal samples were significantly higher than that in the control group (*P* < 0.05). Instead, the relative abundance of *Campylobacterales,Bacillales,Chlamydiales, Pseudomonadales* and *Actinomycetales* in both ZnO and antibiotics-treated ileal samples were significantly lower than that in the control group (*P* < 0.05).

**Table S9.The orders composition of colonic microbiome in each group.**

| **Orders** | **Control group**  **(n=4)** | **Antibiotics group**  **(n=4)** | **ZnO**  **group**  **(n=3)** | **P.value**  **(Antibiotics VS Control)** | **P.value**  **(ZnO VS Control)** | **P.value**  **(ZnO VS Antibiotics)** |
| --- | --- | --- | --- | --- | --- | --- |
|  |  |  |  |  |  |  |
| Clostridiales | 59.07±6.06% | 68.49±7.78% | 53.72±14.51% | 0.10 | 0.53 | 0.14 |
| Bacteroidales | 19.10±7.06% | 13.06±6.16% | 8.96±1.68% | 0.24 | 0.06 | 0.32 |
| Lactobacillales | 6.98±6.29% | 3.25±1.02% | 12.94±10.57% | 0.28 | 0.39 | 0.12 |
| Spirochaetales | 2.65±2.85% | 0.70±0.11% | 0.82±0.20% | 0.22 | 0.33 | 0.33 |
| Erysipelotrichales | 1.99±1.43% | 3.30±3.49% | 2.96±2.25% | 0.52 | 0.52 | 0.89 |
| Turicibacterales | 1.19±0.81% | 2.65±2.73% | 1.20±1.36% | 0.34 | 0.99 | 0.44 |
| RF39 | 1.40±0.46% | 1.30±0.49% | 1.65±0.81% | 0.80 | 0.62 | 0.51 |
| CW040 | 0.48±0.68% | 0.26±0.23% | 2.86±3.94% | 0.57 | 0.27 | 0.23 |
| **Methanobacteriales** | 0.33±0.13% | 0.24±0.18% | 7.02±4.60% | 0.41 | **0.03** | **0.03** |
| **Pasteurellales** | 0.29±0.02% | 0.31±0.07% | 0.23±0.04% | 0.70 | **0.03** | 0.12 |
| Coriobacteriales | 0.20±0.22% | 0.62±1.08% | 0.27±0.16% | 0.48 | 0.67 | 0.61 |
| **Enterobacteriales** | 0.20±0.01% | 0.26±0.02% | 0.22±0.02% | **0.00** | 0.09 | **0.04** |
| YS2 | 0.17±0.12% | 0.07±0.03% | 0.17±0.13% | 0.16 | 0.97 | 0.19 |
| Bifidobacteriales | 0.13±0.18% | 0.05±0.02% | 0.21±0.19% | 0.40 | 0.55 | 0.12 |
| Campylobacterales | 0.11±0.08% | 0.03±0.00% | 0.19±0.21% | 0.07 | 0.51 | 0.17 |
| WCHB1-41 | 0.06±0.02% | 0.06±0.02% | 0.05±0.02% | 0.85 | 0.50 | 0.46 |
| Tremblayales | 0.05±0.03% | 0.05±0.01% | 1.48±2.41% | 0.76 | 0.27 | 0.27 |
| Desulfovibrionales | 0.05±0.05% | 0.02±0.01% | 0.14±0.19% | 0.31 | 0.38 | 0.24 |
| Fibrobacterales | 0.04±0.03% | 0.11±0.19% | 0.02±0.01% | 0.48 | 0.50 | 0.48 |
| Bacillales | 0.02±0.02% | 0.06±0.08% | 0.04±0.05% | 0.31 | 0.47 | 0.65 |
| Verrucomicrobiales | 0.01±0.01% | 0.04±0.04% | 0.17±0.19% | 0.27 | 0.17 | 0.25 |
| Synergistales | 0.01±0.01% | 0.23±0.42% | 0.03±0.03% | 0.34 | 0.27 | 0.46 |
| Unassigned | 5.30±0.47% | 4.71±0.71% | 4.33±0.24% | 0.21 | 0.02 | 0.41 |

The relative abundance oforders higher than 0.05% in any group were shown. The most predominant orders (the average abundance of all samples higher than 10%) including *Clostridiales* and *Bacteroidales* showed no significant differences between two groups.The relative abundance of *Methanobacteriales* in ZnO group was significantly higher than that in the control or antibiotics group in colonic microbiota (*P* < 0.05).It is worthy of highlighting that antibiotics-treated group significantly increased the relative abundance of *Enterobacteriales* when compared with control or ZnO group (*P* < 0.05).

**Table S10.The families composition of ileal microbiome in each group (n=4 per group).**

| **Families** | **Control**  **group** | **Antibiotics group** | **ZnO**  **group** | **P.value**  **(Antibiotics VS Control)** | **P.value**  **(ZnO VS Control)** | **P.value**  **(ZnO VS Antibiotics)** |
| --- | --- | --- | --- | --- | --- | --- |
|  |  |  |  |  |  |  |
| Lactobacillaceae | 40.84±23.73% | 23.77±22.27% | 39.07±31.45% | 0.33 | 0.93 | 0.46 |
| Clostridiaceae | 27.29±20.61% | 36.52±11.82% | 26.87±23.84% | 0.47 | 0.98 | 0.5 |
| Ruminococcaceae | 3.69±0.28% | 3.95±0.23% | 3.68±0.25% | 0.2 | 0.98 | 0.17 |
| **Streptococcaceae** | 3.04±1.07% | 0.51±0.25% | 0.80±0.35% | **0** | **0.01** | 0.23 |
| **Helicobacteraceae** | 2.62±0.64% | 0.00±0.00% | 0.00±0.00% | **0** | **0** | 0.97 |
| **Lachnospiraceae** | 2.25±0.06% | 1.32±0.13% | 1.07±0.20% | **0** | **0** | 0.07 |
| **Paenibacillaceae** | 1.96±0.24% | 0.00±0.00% | 0.00±0.00% | **0** | **0** | 1 |
| Veillonellaceae | 1.19±1.76% | 1.29±0.21% | 3.83±5.15% | 0.92 | 0.37 | 0.36 |
| Peptostreptococcaceae | 1.19±0.79% | 4.48±3.30% | 1.97±2.42% | 0.1 | 0.56 | 0.27 |
| Prevotellaceae | 0.79±0.15% | 0.65±0.16% | 0.77±0.33% | 0.26 | 0.92 | 0.53 |
| Pasteurellaceae | 0.51±0.48% | 2.78±2.87% | 0.84±0.67% | 0.17 | 0.45 | 0.24 |
| Turicibacteraceae | 0.51±0.48% | 3.10±2.77% | 3.03±2.98% | 0.11 | 0.15 | 0.97 |
| [Paraprevotellaceae] | 0.37±0.04% | 0.48±0.01% | 0.44±0.12% | 0 | 0.32 | 0.55 |
| **S24-7** | 0.30±0.03% | 0.40±0.02% | 0.38±0.03% | **0** | **0.01** | 0.23 |
| **Chlamydiaceae** | 0.28±0.03% | 0.00±0.00% | 0.00±0.00% | **0** | **0** | 0.18 |
| Planococcaceae | 0.14±0.07% | 0.03±0.02% | 0.03±0.04% | 0.03 | 0.05 | 0.93 |
| **Bacteroidaceae** | 0.12±0.04% | 0.02±0.00% | 0.01±0.00% | **0** | **0** | 0.05 |
| **Erysipelotrichaceae** | 0.10±0.02% | 0.32±0.03% | 0.34±0.04% | **0** | **0** | 0.29 |
| **Pseudomonadaceae** | 0.10±0.02% | 0.00±0.00% | 0.00±0.00% | **0** | **0** | 0.36 |
| **Bacillaceae** | 0.09±0.03% | 0.01±0.01% | 0.01±0.01% | **0** | **0** | 0.99 |
| **Enterobacteriaceae** | 0.07±0.05% | 0.50±0.23% | 2.69±3.64% | **0.01** | **0.01** | 0.27 |
| **Corynebacteriaceae** | 0.06±0.01% | 0.00±0.00% | 0.00±0.00% | **0** | **0** | 1 |
| Staphylococcaceae | 0.06±0.05% | 0.00±0.00% | 0.01±0.01% | 0.06 | 0.07 | 0.44 |
| [Mogibacteriaceae] | 0.05±0.02% | 0.03±0.01% | 0.04±0.01% | 0.22 | 0.22 | 0.9 |
| **Porphyromonadaceae** | 0.04±0.01% | 0.09±0.02% | 0.07±0.01% | **0.01** | **0** | 0.17 |
| Coriobacteriaceae | 0.03±0.01% | 0.05±0.03% | 0.06±0.04% | 0.14 | 0.16 | 0.66 |
| Bifidobacteriaceae | 0.01±0.00% | 0.13±0.13% | 1.22±2.04% | 0.13 | 0.28 | 0.32 |
| **Methanobacteriaceae** | 0.00±0.00% | 0.08±0.01% | 0.08±0.02% | **0** | **0** | 0.49 |
| **Spirochaetaceae** | 0.00±0.00% | 0.76±0.04% | 0.74±0.16% | **0** | **0** | 0.84 |
| **F16** | 0.00±0.00% | 0.07±0.02% | 0.05±0.02% | **0** | **0.01** | 0.14 |
| **Christensenellaceae** | 0.00±0.00% | 0.06±0.02% | 0.05±0.01% | **0** | **0** | 0.35 |
| Unassigned | 8.95±1.74% | 6.48±0.72% | 5.65±1.03% | 0.04 | 0.02 | 0.23 |

The relative abundance of families higher than 0.05% in any group were shown. The most predominant families (the average abundance of all samples higher than 10%) including *Lactobacillaceae* and *Clostridiaceae* showed no significant differences between two groups. The proportion of *S24-7,Erysipelotrichaceae,Enterobacteriaceae, Porphyromonadaceae,Methanobacteriaceae, Spirochaetaceae, F16* and *Christensenellaceae* in both ZnO and antibiotics-treated ileal samples were significantly higher than that in the control group (*P* < 0.05). Instead, the relative abundance of *Streptococcaceae,Helicobacteraceae,Lachnospiraceae, Paenibacillaceae, Chlamydiaceae, Bacteroidaceae,Pseudomonadaceae,Bacillaceae* and *Corynebacteriaceae* in both ZnO and antibiotics-treated ileal samples were significantly lower than that in the control group (*P* < 0.05).

**Table S11.The families composition of colonic microbiome in each group.**

| **Families** | **Control group**  **(n=4)** | **Antibiotics group**  **(n=4)** | **ZnO**  **group**  **(n=3)** | **P.value**  **(Antibiotics VS Control)** | **P.value**  **(ZnO VS Control)** | **P.value**  **(ZnO VS Antibiotics)** |
| --- | --- | --- | --- | --- | --- | --- |
|  |  |  |  |  |  |  |
| Ruminococcaceae | 22.10±8.01% | 29.93±8.54% | 28.86±13.88% | 0.23 | 0.45 | 0.9 |
| Clostridiaceae | 9.41±4.55% | 7.85±3.19% | 5.85±0.97% | 0.6 | 0.25 | 0.35 |
| Prevotellaceae | 9.02±6.86% | 5.68±2.48% | 3.31±1.35% | 0.39 | 0.22 | 0.2 |
| Lactobacillaceae | 6.54±6.18% | 3.06±1.00% | 12.38±10.05% | 0.31 | 0.38 | 0.11 |
| [Paraprevotellaceae] | 4.53±4.55% | 1.70±0.47% | 2.51±1.09% | 0.26 | 0.49 | 0.23 |
| **Lachnospiraceae** | 4.42±0.44% | 2.19±0.68% | 3.18±1.18% | **0** | 0.11 | 0.21 |
| Veillonellaceae | 3.39±2.24% | 8.99±9.33% | 2.42±1.24% | 0.29 | 0.53 | 0.29 |
| Spirochaetaceae | 2.65±2.85% | 0.70±0.11% | 0.82±0.20% | 0.22 | 0.33 | 0.33 |
| Peptostreptococcaceae | 2.41±1.58% | 1.84±0.73% | 0.66±0.43% | 0.53 | 0.13 | 0.06 |
| Erysipelotrichaceae | 1.99±1.43% | 3.30±3.49% | 2.96±2.25% | 0.52 | 0.52 | 0.89 |
| S24-7 | 1.96±0.56% | 1.37±0.56% | 1.27±0.39% | 0.19 | 0.13 | 0.82 |
| Turicibacteraceae | 1.19±0.81% | 2.65±2.73% | 1.20±1.36% | 0.34 | 0.99 | 0.44 |
| p-2534-18B5 | 0.82±1.53% | 0.04±0.03% | 0.06±0.08% | 0.35 | 0.44 | 0.69 |
| Porphyromonadaceae | 0.66±0.26% | 3.06±4.79% | 0.44±0.23% | 0.36 | 0.29 | 0.4 |
| F16 | 0.48±0.68% | 0.26±0.23% | 2.86±3.94% | 0.57 | 0.27 | 0.23 |
| Streptococcaceae | 0.43±0.40% | 0.18±0.04% | 0.55±0.57% | 0.25 | 0.76 | 0.24 |
| **Methanobacteriaceae** | 0.33±0.13% | 0.24±0.18% | 7.02±4.60% | 0.41 | **0.03** | **0.03** |
| **Pasteurellaceae** | 0.29±0.02% | 0.31±0.07% | 0.23±0.04% | 0.7 | **0.03** | 0.12 |
| Coriobacteriaceae | 0.20±0.22% | 0.62±1.08% | 0.27±0.16% | 0.48 | 0.67 | 0.61 |
| **Enterobacteriaceae** | 0.20±0.01% | 0.26±0.02% | 0.22±0.02% | **0** | 0.09 | **0.04** |
| Christensenellaceae | 0.16±0.12% | 0.08±0.04% | 0.13±0.07% | 0.24 | 0.7 | 0.32 |
| Bifidobacteriaceae | 0.13±0.18% | 0.05±0.02% | 0.21±0.19% | 0.4 | 0.55 | 0.12 |
| Rikenellaceae | 0.11±0.21% | 0.00±0.00% | 0.00±0.00% | 0.35 | 0.44 | 0.44 |
| Bacteroidaceae | 0.11±0.12% | 0.03±0.02% | 0.02±0.00% | 0.27 | 0.28 | 0.41 |
| Campylobacteraceae | 0.10±0.07% | 0.02±0.00% | 0.14±0.16% | 0.06 | 0.64 | 0.17 |
| [Mogibacteriaceae] | 0.09±0.07% | 0.05±0.02% | 0.11±0.06% | 0.29 | 0.62 | 0.08 |
| Dehalobacteriaceae | 0.06±0.07% | 0.10±0.13% | 0.03±0.02% | 0.64 | 0.42 | 0.4 |
| Desulfovibrionaceae | 0.05±0.05% | 0.02±0.01% | 0.14±0.19% | 0.31 | 0.38 | 0.24 |
| RF16 | 0.05±0.03% | 0.08±0.11% | 0.07±0.04% | 0.64 | 0.49 | 0.91 |
| Succinivibrionaceae | 0.05±0.02% | 0.03±0.02% | 0.04±0.02% | 0.33 | 0.49 | 0.8 |
| RFP12 | 0.05±0.01% | 0.05±0.02% | 0.04±0.02% | 0.61 | 0.9 | 0.64 |
| Fibrobacteraceae | 0.04±0.03% | 0.11±0.19% | 0.02±0.01% | 0.48 | 0.5 | 0.48 |
| Peptococcaceae | 0.01±0.01% | 0.01±0.00% | 0.05±0.04% | 0.54 | 0.21 | 0.16 |
| Verrucomicrobiaceae | 0.01±0.01% | 0.04±0.04% | 0.16±0.19% | 0.27 | 0.17 | 0.25 |
| Bacillaceae | 0.01±0.01% | 0.05±0.07% | 0.03±0.04% | 0.3 | 0.4 | 0.66 |
| Dethiosulfovibrionaceae | 0.01±0.01% | 0.23±0.42% | 0.03±0.03% | 0.34 | 0.27 | 0.46 |
| Unassigned | 5.30±0.47% | 4.71±0.71% | 4.33±0.24% | 0.21 | 0.02 | 0.41 |

The relative abundance of families higher than 0.05% in any group were shown. The most predominant family (the average abundance of all samples higher than 10%) *Ruminococcaceae* showed no significant differences between two groups. The relative abundance of *Methanobacteriaceae* in ZnO group was significantly higher than that in the control or antibiotics group in colonic microbiota (*P* < 0.05).It is worthy of highlighting that antibiotics-treated group significantly increased the relative abundance of *Enterobacteriaceae* when compared with control or ZnO group (*P* < 0.05).

**Table S12.The genera composition of ileal microbiome in each group (n=4 per group).**

| **Genera** | **Control**  **group** | **Antibiotics**  **group** | **ZnO**  **group** | **P.value**  **(Antibiotics VS Control)** | **P.value**  **(ZnO VS Control)** | **P.value**  **(ZnO VS Antibiotics)** |
| --- | --- | --- | --- | --- | --- | --- |
|  |  |  |  |  |  |  |
| Lactobacillus | 40.84±23.72% | 23.77±22.27% | 39.07±31.45% | 0.33 | 0.93 | 0.46 |
| Clostridium | 4.07±4.62% | 2.90±2.34% | 1.59±1.13% | 0.67 | 0.34 | 0.35 |
| **Streptococcus** | 2.97±1.04% | 0.46±0.26% | 0.75±0.36% | **0** | **0.01** | 0.25 |
| **Aneurinibacillus** | 1.86±0.25% | 0.00±0.00% | 0.00±0.00% | **0** | **0** | 1 |
| **Helicobacter** | 1.80±0.41% | 0.00±0.00% | 0.00±0.00% | **0** | **0** | 0.97 |
| Veillonella | 0.94±1.73% | 0.20±0.10% | 1.37±2.21% | 0.43 | 0.77 | 0.33 |
| Prevotella | 0.79±0.15% | 0.65±0.16% | 0.77±0.33% | 0.26 | 0.93 | 0.53 |
| **Oscillospira** | 0.67±0.04% | 0.25±0.05% | 0.24±0.04% | **0** | **0** | 0.62 |
| **Flexispira** | 0.67±0.19% | 0.00±0.00% | 0.00±0.00% | **0** | **0** | 1 |
| SMB53 | 0.65±0.35% | 0.61±0.24% | 0.42±0.08% | 0.85 | 0.23 | 0.17 |
| **Blautia** | 0.57±0.03% | 0.04±0.02% | 0.03±0.00% | **0** | **0** | 0.74 |
| Turicibacter | 0.51±0.48% | 3.10±2.77% | 3.03±2.98% | 0.11 | 0.15 | 0.97 |
| Actinobacillus | 0.46±0.44% | 2.05±1.95% | 0.70±0.58% | 0.16 | 0.54 | 0.23 |
| **Dorea** | 0.29±0.02% | 0.08±0.02% | 0.06±0.01% | **0** | **0** | 0.14 |
| **Coprococcus** | 0.28±0.12% | 0.13±0.02% | 0.11±0.05% | **0.04** | **0.03** | 0.47 |
| **Chlamydia** | 0.25±0.03% | 0.00±0.00% | 0.00±0.00% | **0** | **0** | 0.18 |
| **[Prevotella]** | 0.24±0.02% | 0.20±0.02% | 0.17±0.05% | **0.03** | **0.03** | 0.34 |
| **Phascolarctobacterium** | 0.24±0.04% | 0.39±0.04% | 0.40±0.07% | **0** | **0.01** | 0.8 |
| Ruminococcus | 0.19±0.02% | 0.20±0.02% | 0.23±0.06% | 0.6 | 0.3 | 0.4 |
| Faecalibacterium | 0.14±0.03% | 0.07±0.03% | 0.09±0.04% | 0.01 | 0.11 | 0.37 |
| Sarcina | 0.13±0.17% | 0.13±0.05% | 0.14±0.14% | 0.98 | 0.93 | 0.91 |
| **CF231** | 0.12±0.02% | 0.24±0.03% | 0.24±0.09% | **0** | **0.04** | 0.99 |
| **Bacteroides** | 0.12±0.04% | 0.02±0.00% | 0.01±0.00% | **0** | **0** | 0.05 |
| **Roseburia** | 0.10±0.02% | 0.04±0.02% | 0.02±0.01% | **0** | **0** | 0.09 |
| **Sporosarcina** | 0.10±0.02% | 0.00±0.00% | 0.00±0.00% | **0** | **0** | 1 |
| **Pseudomonas** | 0.09±0.01% | 0.00±0.00% | 0.00±0.00% | **0** | **0** | 0.36 |
| **Corynebacterium** | 0.06±0.01% | 0.00±0.00% | 0.00±0.00% | **0** | **0** | 1 |
| Staphylococcus | 0.06±0.05% | 0.00±0.00% | 0.01±0.01% | 0.06 | 0.07 | 0.44 |
| **[Ruminococcus]** | 0.05±0.01% | 0.02±0.01% | 0.02±0.01% | **0** | **0** | 0.91 |
| **Parabacteroides** | 0.04±0.01% | 0.09±0.02% | 0.07±0.01% | **0.01** | **0** | 0.18 |
| **[Eubacterium]** | 0.04±0.02% | 0.13±0.03% | 0.15±0.03% | **0** | **0** | 0.42 |
| Aggregatibacter | 0.03±0.05% | 0.32±0.54% | 0.02±0.02% | 0.32 | 0.76 | 0.31 |
| Bifidobacterium | 0.01±0.00% | 0.13±0.13% | 1.21±2.01% | 0.13 | 0.28 | 0.32 |
| **Lachnospira** | 0.01±0.00% | 0.15±0.05% | 0.13±0.05% | **0** | **0** | 0.63 |
| **Bulleidia** | 0.01±0.00% | 0.06±0.02% | 0.05±0.02% | **0** | **0** | 0.52 |
| **Treponema** | 0.00±0.00% | 0.76±0.04% | 0.74±0.16% | **0** | **0** | 0.84 |
| **Methanobrevibacter** | 0.00±0.00% | 0.06±0.01% | 0.08±0.02% | **0** | **0** | 0.24 |
| **p-75-a5** | 0.00±0.00% | 0.11±0.02% | 0.10±0.02% | **0** | **0** | 0.72 |
| **Gallibacterium** | 0.00±0.00% | 0.09±0.01% | 0.10±0.06% | **0** | **0.02** | 0.77 |
| **Anaerovibrio** | 0.00±0.00% | 0.06±0.02% | 0.03±0.01% | **0** | **0.01** | 0.06 |
| Megasphaera | 0.00±0.00% | 0.20±0.06% | 1.43±2.46% | 0 | 0.29 | 0.36 |
| Mitsuokella | 0.00±0.00% | 0.02±0.01% | 0.07±0.12% | 0.02 | 0.29 | 0.42 |
| Dialister | 0.00±0.00% | 0.02±0.00% | 0.12±0.17% | 0 | 0.23 | 0.32 |
| Unassigned | 8.95±1.74% | 6.48±0.72% | 5.65±1.03% | 0.04 | 0.02 | 0.23 |

The relative abundance ofgenera higher than 0.05% in any group were shown. The most predominant genus (the average abundance of all samples higher than 10%) *Lactobacillus* showed no significant differences between two groups. The proportion of *Phascolarctobacterium,CF231,Parabacteroides, [Eubacterium],Lachnospira, Bulleidia, Treponema, Methanobrevibacter, p-75-a5,Gallibacterium* and *Anaerovibrio* in both ZnO and antibiotics-treated ileal samples were significantly higher than that in the control group (*P* < 0.05). Instead, the relative abundance of *Streptococcus,Aneurinibacillus, Helicobacter, Oscillospira, Flexispira, Blautia, Dorea, Coprococcus, Chlamydia, [Prevotella],Bacteroides,Roseburia, Sporosarcina,Pseudomonas,Corynebacterium* and*[Ruminococcus]*in both ZnO and antibiotics-treated ileal samples were significantly lower than that in the control group (*P* < 0.05).

**Table S13.The genera composition of colonic microbiome in each group.**

| **Genera** | **Control**  **group**  **(n=4)** | **Antibiotics group**  **(n=4)** | **ZnO**  **group**  **(n=3)** | **P.value**  **(Antibiotics VS Control)** | **P.value**  **(ZnO VS Control)** | **P.value**  **(ZnO VS Antibiotics)** |
| --- | --- | --- | --- | --- | --- | --- |
|  |  |  |  |  |  |  |
| Prevotella | 9.01±6.87% | 5.67±2.47% | 3.30±1.35% | 0.4 | 0.22 | 0.2 |
| Lactobacillus | 6.54±6.18% | 3.06±1.00% | 12.38±10.05% | 0.31 | 0.38 | 0.11 |
| [Prevotella] | 3.61±3.84% | 1.13±0.67% | 1.75±0.83% | 0.25 | 0.46 | 0.32 |
| Treponema | 2.65±2.85% | 0.70±0.11% | 0.82±0.20% | 0.22 | 0.33 | 0.33 |
| Turicibacter | 1.19±0.81% | 2.65±2.73% | 1.20±1.36% | 0.34 | 0.99 | 0.44 |
| Clostridium | 1.04±0.10% | 1.17±0.59% | 0.82±0.22% | 0.66 | 0.14 | 0.38 |
| p-75-a5 | 1.01±1.03% | 1.02±1.42% | 0.42±0.35% | 1 | 0.39 | 0.51 |
| Phascolarctobacterium | 0.96±0.34% | 0.51±0.29% | 0.48±0.14% | 0.09 | 0.07 | 0.86 |
| Oscillospira | 0.92±0.35% | 1.94±1.59% | 0.81±0.17% | 0.26 | 0.64 | 0.29 |
| Ruminococcus | 0.83±0.28% | 1.39±1.72% | 1.28±0.73% | 0.54 | 0.3 | 0.93 |
| CF231 | 0.66±0.42% | 0.40±0.18% | 0.64±0.27% | 0.29 | 0.95 | 0.21 |
| Parabacteroides | 0.65±0.26% | 3.06±4.79% | 0.44±0.23% | 0.35 | 0.32 | 0.4 |
| SMB53 | 0.57±0.28% | 0.55±0.18% | 0.31±0.15% | 0.87 | 0.21 | 0.13 |
| Megasphaera | 0.57±0.72% | 0.31±0.17% | 0.77±0.91% | 0.51 | 0.76 | 0.36 |
| Lachnospira | 0.50±0.13% | 0.30±0.14% | 0.30±0.15% | 0.08 | 0.12 | 0.97 |
| [Eubacterium] | 0.46±0.27% | 1.90±3.11% | 1.42±1.57% | 0.39 | 0.27 | 0.82 |
| Bulleidia | 0.45±0.55% | 0.26±0.17% | 1.06±1.06% | 0.54 | 0.36 | 0.18 |
| Streptococcus | 0.43±0.40% | 0.18±0.04% | 0.53±0.56% | 0.26 | 0.78 | 0.25 |
| Veillonella | 0.29±0.29% | 0.12±0.02% | 0.31±0.32% | 0.28 | 0.93 | 0.27 |
| **Coprococcus** | 0.28±0.09% | 0.13±0.04% | 0.22±0.12% | **0.02** | 0.46 | 0.23 |
| Blautia | 0.26±0.32% | 0.08±0.05% | 0.40±0.33% | 0.31 | 0.61 | 0.11 |
| Dorea | 0.26±0.08% | 0.13±0.07% | 0.28±0.22% | 0.05 | 0.84 | 0.23 |
| **Methanobrevibacter** | 0.25±0.12% | 0.22±0.18% | 5.90±4.11% | 0.79 | **0.04** | **0.04** |
| Actinobacillus | 0.21±0.03% | 0.21±0.05% | 0.17±0.02% | 0.99 | 0.09 | 0.21 |
| YRC22 | 0.17±0.27% | 0.05±0.05% | 0.05±0.05% | 0.43 | 0.5 | 0.97 |
| Faecalibacterium | 0.15±0.05% | 0.52±0.75% | 0.37±0.28% | 0.36 | 0.17 | 0.77 |
| Dialister | 0.13±0.21% | 0.45±0.49% | 0.22±0.31% | 0.27 | 0.64 | 0.51 |
| Bifidobacterium | 0.13±0.18% | 0.05±0.02% | 0.21±0.19% | 0.41 | 0.56 | 0.12 |
| Bacteroides | 0.11±0.12% | 0.03±0.02% | 0.02±0.00% | 0.27 | 0.28 | 0.41 |
| Campylobacter | 0.10±0.07% | 0.02±0.00% | 0.14±0.16% | 0.06 | 0.64 | 0.17 |
| Methanosphaera | 0.08±0.15% | 0.02±0.00% | 1.11±0.95% | 0.41 | 0.08 | 0.06 |
| Acidaminococcus | 0.08±0.11% | 0.05±0.04% | 0.02±0.01% | 0.69 | 0.39 | 0.16 |
| Anaerovibrio | 0.07±0.03% | 0.09±0.05% | 0.03±0.01% | 0.48 | 0.14 | 0.11 |
| **Gallibacterium** | 0.07±0.01% | 0.07±0.02% | 0.04±0.02% | 0.55 | 0.11 | **0.04** |
| Dehalobacterium | 0.06±0.07% | 0.10±0.13% | 0.03±0.02% | 0.64 | 0.42 | 0.4 |
| [Ruminococcus] | 0.06±0.02% | 0.07±0.03% | 0.19±0.11% | 0.57 | 0.07 | 0.09 |
| Roseburia | 0.06±0.03% | 0.05±0.03% | 0.03±0.00% | 0.95 | 0.19 | 0.24 |
| Desulfovibrio | 0.05±0.05% | 0.02±0.01% | 0.14±0.18% | 0.31 | 0.36 | 0.24 |
| Collinsella | 0.05±0.04% | 0.03±0.03% | 0.08±0.07% | 0.53 | 0.49 | 0.25 |
| Fibrobacter | 0.04±0.03% | 0.11±0.19% | 0.02±0.01% | 0.48 | 0.5 | 0.48 |
| Peptococcus | 0.01±0.01% | 0.01±0.00% | 0.05±0.04% | 0.54 | 0.21 | 0.16 |
| Akkermansia | 0.01±0.01% | 0.04±0.04% | 0.16±0.19% | 0.27 | 0.17 | 0.25 |
| Pyramidobacter | 0.01±0.00% | 0.17±0.32% | 0.02±0.03% | 0.34 | 0.23 | 0.47 |
| Unassigned | 5.30±0.47% | 4.71±0.71% | 4.33±0.24% | 0.21 | 0.02 | 0.41 |

The relative abundance of genera higher than 0.05% in any group were shown. The most predominant genera (the average abundance of all samples higher than 1%) including *Prevotella* and *Lactobacillus* showed no significant differences between two groups. The relative abundance of *Methanobrevibacter* in ZnO group was significantly higher than that in the control or antibiotics group in colonic microbiota (*P* < 0.05).

**Table S14.The significant differences of ileal strains(n=4 per group).**

| **Bacterial strains in the ileum** | **Control**  **group**  **(%)** | **ZnO**  **group**  **(%)** | **Antibiotics**  **group**  **(%)** | **P.value (Tukey-Kramer ANOVA)** | **P.value (ZnO vs Control)** | **P.value (Antibiotics vs Control)** | **P.value (ZnO vs Antibiotics)** |
| --- | --- | --- | --- | --- | --- | --- | --- |
| **Increased by ZnO or antibiotics** | | | | | | | |
| *Clostridium cellulolyticum H10* | 0.003 ±0.003 | 0.072 ±0.039 | 0.085 ±0.019 | 0.01 | 0.02 | 0 | 0.61 |
| *Clostridium phytofermentans ISDg* | 0.003 ±0.002 | 0.052 ±0.022 | 0.096 ±0.044 | 0.01 | 0.01 | 0.01 | 0.16 |
| *Clostridium saccharolyticum WM1* | 0.307 ±0.015 | 0.712 ±0.218 | 0.898 ±0.210 | 0.01 | 0.02 | 0 | 0.33 |
| *Clostridium thermocellum DSM 1313* | 0.027 ±0.005 | 0.139 ±0.036 | 0.210 ±0.039 | 0 | 0 | 0 | 0.06 |
| *Coprococcus sp. ART55/1* | 0.057 ±0.013 | 0.112 ±0.024 | 0.112 ±0.019 | 0.01 | 0.01 | 0.01 | 0.98 |
| *Ethanoligenens harbinense YUAN-3* | 0.058 ±0.012 | 0.123 ±0.033 | 0.115 ±0.005 | 0.01 | 0.02 | 0 | 0.7 |
| *Eubacterium eligens ATCC 27750* | 0.023 ±0.004 | 0.178 ±0.068 | 0.229 ±0.068 | 0 | 0.01 | 0 | 0.39 |
| *Gallibacterium anatis UMN179* | 0.000 ±0.000 | 0.154 ±0.086 | 0.150 ±0.029 | 0.01 | 0.02 | 0 | 0.94 |
| *Lactobacillus helveticus CNRZ32* | 0.111 ±0.029 | 0.249 ±0.082 | 0.367 ±0.077 | 0 | 0.03 | 0 | 0.12 |
| *Methanobrevibacter smithii ATCC 35061* | 0.000 ±0.001 | 0.079 ±0.015 | 0.068 ±0.013 | 0 | 0 | 0 | 0.4 |
| *Oscillibacter valericigenes Sjm18-20* | 0.265 ±0.023 | 1.037 ±0.195 | 1.186 ±0.202 | 0 | 0 | 0 | 0.39 |
| *Roseburia intestinalis XB6B4* | 0.056 ±0.017 | 0.105 ±0.028 | 0.120 ±0.011 | 0.01 | 0.04 | 0 | 0.43 |
| *Selenomonas ruminantium subsp. lactilytica TAM6421* | 0.000 ±0.001 | 0.441 ±0.144 | 0.433 ±0.040 | 0 | 0 | 0 | 0.93 |
| *Treponema brennaborense DSM 12168* | 0.001 ±0.002 | 0.880 ±0.213 | 0.905 ±0.058 | 0 | 0 | 0 | 0.85 |
| *Treponema succinifaciens DSM 2489* | 0.000 ±0.000 | 0.049 ±0.011 | 0.069 ±0.008 | 0 | 0 | 0 | 0.04 |
| **Decreased by ZnO or antibiotics** | | | | | | | |
| *Anoxybacillus flavithermus WK1* | 0.117 ±0.018 | 0.000 ±0.000 | 0.001 ±0.002 | 0 | 0 | 0 | 0.36 |
| *Bacteroides helcogenes P 36-108* | 0.061 ±0.013 | 0.004 ±0.005 | 0.009 ±0.003 | 0 | 0 | 0 | 0.22 |
| *Bacteroides thetaiotaomicron VPI-5482* | 0.095 ±0.011 | 0.009 ±0.003 | 0.007 ±0.005 | 0 | 0 | 0 | 0.68 |
| *Bacteroides vulgatus ATCC 8482* | 0.061 ±0.009 | 0.002 ±0.003 | 0.002 ±0.002 | 0 | 0 | 0 | 0.87 |
| *Chlamydia trachomatis RC-J(s)/122* | 0.435 ±0.042 | 0.001 ±0.002 | 0.003 ±0.002 | 0 | 0 | 0 | 0.16 |
| *Clostridium pasteurianum BC1* | 0.054 ±0.024 | 0.004 ±0.005 | 0.005 ±0.004 | 0 | 0.01 | 0.01 | 0.81 |
| *Coprococcus catus GD/7* | 0.240 ±0.055 | 0.047 ±0.027 | 0.062 ±0.010 | 0 | 0 | 0 | 0.41 |
| *Corynebacterium glutamicum MB001* | 0.074 ±0.012 | 0.000 ±0.000 | 0.000 ±0.000 | 0 | 0 | 0 | 1 |
| *Faecalibacterium prausnitzii SL3/3* | 1.007 ±0.198 | 0.515 ±0.095 | 0.466 ±0.078 | 0 | 0.01 | 0 | 0.52 |
| *Geobacillus sp. JF8* | 1.379 ±0.097 | 0.000 ±0.000 | 0.000 ±0.000 | 0 | 0 | 0 | 1 |
| *Helicobacter cetorum MIT 99-5656* | 0.080 ±0.025 | 0.000 ±0.000 | 0.000 ±0.000 | 0 | 0 | 0 | 1 |
| *Helicobacter hepaticus ATCC 51449* | 1.875 ±0.482 | 0.002 ±0.003 | 0.002 ±0.002 | 0 | 0 | 0 | 0.89 |
| *Helicobacter pylori F30* | 0.750 ±0.213 | 0.000 ±0.000 | 0.000 ±0.000 | 0 | 0 | 0 | 1 |
| *Helicobacter pylori SouthAfrica20* | 0.094 ±0.034 | 0.000 ±0.000 | 0.000 ±0.000 | 0 | 0 | 0 | 1 |
| *Helicobacter pylori XZ274* | 0.061 ±0.025 | 0.000 ±0.000 | 0.000 ±0.000 | 0 | 0.01 | 0.01 | 1 |
| *Pseudomonas fluorescens SBW25* | 0.059 ±0.011 | 0.000 ±0.000 | 0.000 ±0.000 | 0 | 0 | 0 | 1 |
| *Roseburia hominis A2-183* | 0.259 ±0.064 | 0.048 ±0.015 | 0.048 ±0.009 | 0 | 0 | 0 | 0.93 |
| *Ruminococcus albus 7* | 0.126 ±0.024 | 0.043 ±0.011 | 0.051 ±0.010 | 0 | 0 | 0 | 0.41 |
| *Ruminococcus obeum A2-162* | 0.741 ±0.076 | 0.037 ±0.012 | 0.057 ±0.018 | 0 | 0 | 0 | 0.16 |
| *Ruminococcus sp. SR1/5* | 0.340 ±0.095 | 0.044 ±0.007 | 0.072 ±0.021 | 0 | 0 | 0 | 0.07 |
| *Streptococcus pasteurianus ATCC 43144* | 3.056 ±0.874 | 0.579 ±0.131 | 0.499 ±0.246 | 0 | 0 | 0 | 0.63 |
| *Streptococcus thermophilus ND03* | 0.105 ±0.041 | 0.029 ±0.019 | 0.022 ±0.004 | 0.01 | 0.03 | 0.01 | 0.56 |

The relative abundance (higher than 0.05%) of about 15 bacterial strains of ileal microbiota were both significantly increased in ZnO group or antibiotics group, such as 4 *Clostridium* strains, 2 *Treponema* strains,1 *Lactobacillus* strain, 1 *Eubacterium* strain, 1 *Methanobrevibacter* strain, 1 *Selenomonas* strain and so on (*P*<0.05). Meanwhile, the relative abundance (higher than 0.05%) of about 22 bacterial strains of ileal microbiota were both significantly reduced in ZnO group or antibiotics group, such as 5 *Helicobacter* strains, 3 *Ruminococcus* strains, 3 *Bacteroides* strains, 2 *Streptococcus* strains, 1 *Chlamydia* strain,1 *Pseudomonas* strain and so on (*P*<0.05).

**Table S15.The significant differences of colonic strains.**

| **Strains** | **Control**  **group**  **(n=4)** | **ZnO**  **group**  **(n=3)** | **Antibiotics**  **group**  **(n=4)** | **P.value**  **(Tukey-Kramer ANOVA)** | **P.value (Antibiotics vs Control)** | **P.value (ZnO vs Control)** | **P.value (ZnO vs Antibiotics)** |
| --- | --- | --- | --- | --- | --- | --- | --- |
| *Methanobrevibacter smithii ATCC 35061* | 0.40 ±0.16 | 8.41 ±3.57 | 0.39 ±0.28 | **0.00** | 0.97 | **0.01** | **0.01** |
| *Prevotella dentalis DSM 3688* | 3.73 ±0.51 | 1.94 ±0.48 | 2.61 ±0.88 | **0.04** | 0.11 | **0.01** | 0.36 |
| *Eubacterium rectale M104/1* | 2.66 ±0.60 | 0.94 ±0.01 | 0.92 ±0.37 | **0.00** | **0.01** | **0.01** | 0.95 |

The relative abundance of *Methanobrevibacter smithii* was significantly increased 20-fold in ZnO group when compared with control and antibiotics group (*P*<0.05).

**
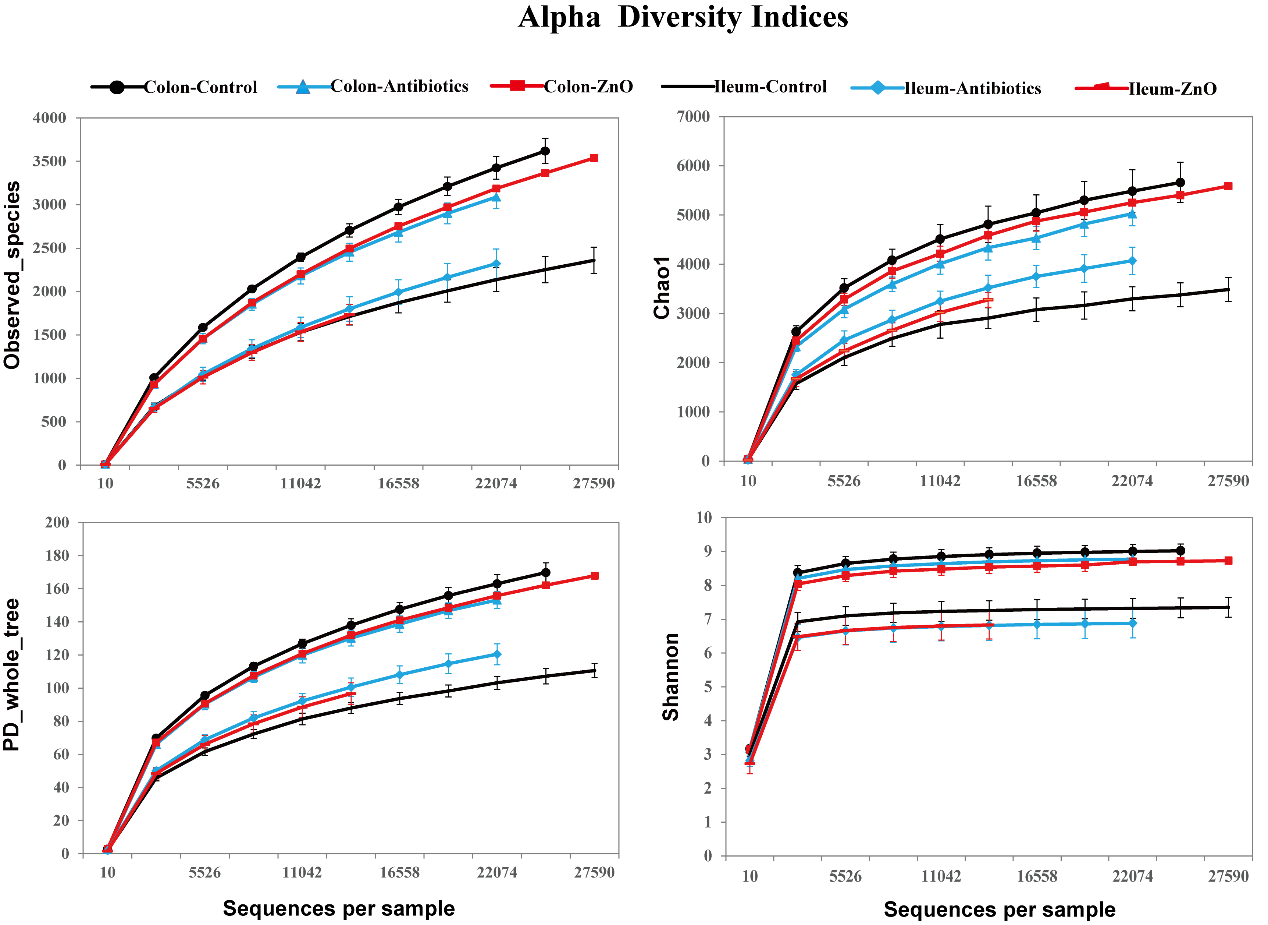
**

**Figure S1| Alpha diversity.** Richness indice (Chao1) and diversity indice (PD whole tree) showed that ZnO group or antibiotics group significantly increased the ileal microbial diversity (*P*<0.05). Richness indices (observed species and Chao1) and diversity indice (PD whole tree) shown that zinc oxide (ZnO) group or antibiotics group significantly decreased the colonic microbial diversity (*P*<0.05).

**
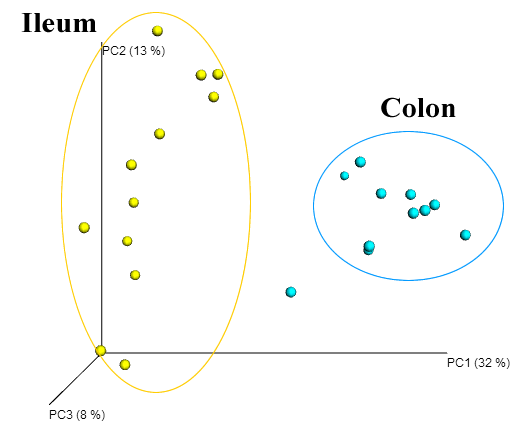
**

**Figure S2 | PCoA analysis.** The principal coordinates analysis (PCoA) of UniFrac distance matrices indicated that it was distinctly difference in distribution of microbiota at ileal and colonic contents.

**

**

**Figure S3 | The bacterial composition in the colon.** From phylum to genus, Some bacterial compositions were significantly affected by high dietary ZnO or antibiotics supplement (*P*<0.05).


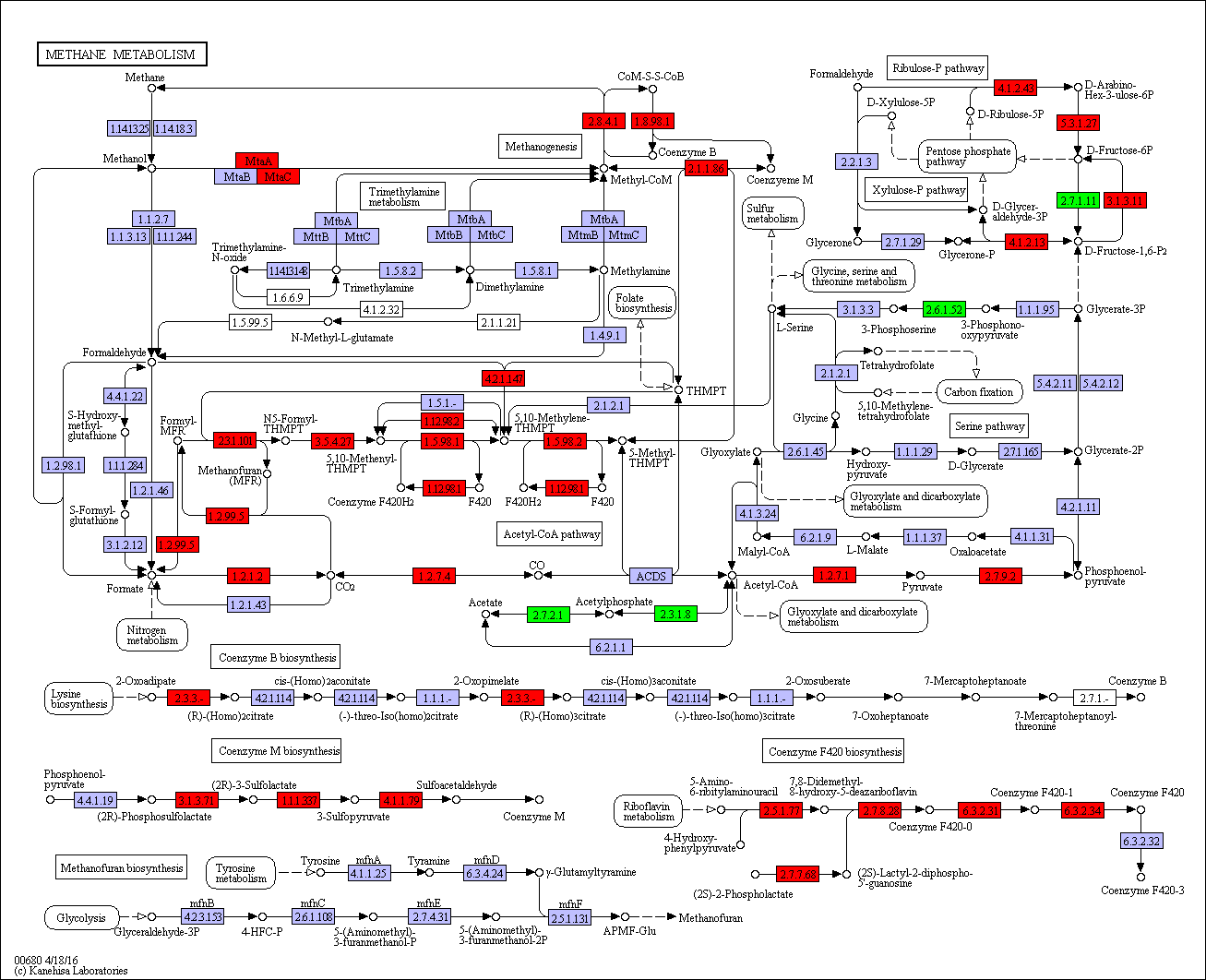


**Figure S4 | Methane metabolism pathway.** The KO terms of methyl-coenzyme M reductase (Mcr, E2.8.4.1) and heterodisulfide reductase (Hdr, E1.8.98.1) were the key enzymes that produced methane, and related with ATP synthesis and energy storage, and were significantly increased by high zinc supplement when compared with control or antibiotics (*P*<0.05*)*.
